# Proteomic analysis of chicken embryo fibroblast cells infected with recombinant H5N1 avian influenza viruses with and without NS1 eIF4GI binding domain

**DOI:** 10.18632/oncotarget.23615

**Published:** 2017-12-22

**Authors:** Kelei Guo, Xian Lin, Yongtao Li, Wei Qian, Zhong Zou, Huanchun Chen, Hongbo Zhou, Meilin Jin

**Affiliations:** ^1^ State Key Laboratory of Agriculture Microbiology, Huazhong Agricultural University, Wuhan, 430070, P. R. China; ^2^ Laboratory of Animal Virology, College of Veterinary Medicine, Huazhong Agricultural University, Wuhan, 430070, P. R. China; ^3^ Key Laboratory of Development of Veterinary Diagnostic Products, Ministry of Agriculture, Huazhong Agricultural University, Wuhan, 430070, P. R. China; ^4^ The Cooperative Innovation Center for Sustainable Pig Production, Wuhan, 430070, P. R. China; ^5^College of Animal Husbandry and Veterinary Science, Henan Agricultural University, Zhengzhou, 450002, P. R. China

**Keywords:** H5N1 avian influenza viruses, NS1 eIF4GI binding domain, proteomic analysis, chicken embryo fibroblast, differentially expressed proteins

## Abstract

Non-structural 1 (NS1) protein is a key virulence factor that regulates replication of influenza virus. A recombinant H5N1 virus lacking the eIF4GI-binding domain of NS1 (rNS1-SD30) exhibits significantly lower pathogenicity than H5N1 virus with an intact eIF4GI-binding domain (rNS1-wt). To further investigate this phenomenon, we performed comparative proteomics analyses to profile host proteins in chicken embryo fibroblasts (CEFs) infected with rNS1-wt and rNS1-SD30 viruses. In total, 81 differentially expressed (DE) proteins were identified at 12, 24, and 36 h post-infection. These proteins are mainly involved in the cytoskeletal, apoptotic and stress responses, transcription regulation, transport and metabolic processes, mRNA processing and splicing, and cellular signal transduction. Overexpression of DE proteins revealed that ANXA7 suppresses propagation of rNS1-SD30, but not rNS1-wt viruses. Moreover, ALDH7A1, ANXA7, and DCTN2 strongly enhanced IFN-β promoter activity induced by chicken MDA5 (chMDA5), and in the case of ANXA7, also by the rNS1-SD30 viral strain. NS1-wt co-transfection suppressed the ANXA7-mediated increase in IFN-β promoter activity induced by chMDA5. These findings highlight the role of NS1 eIF4GI binding domain in H5N1 pathogenicity, and may contribute to the design of antiviral strategies to reduce the high morbidity and mortality associated with this pathogen.

## INTRODUCTION

Influenza A viruses (IAVs) are highly infectious respiratory pathogens in humans and several avian and mammalian species, and are responsible for major pandemics and frequent epidemics associated with high mortality rates [1]. The NS1 protein of IAVs is a non-structural protein, expressed in infected cells, that accumulates in the nucleus early after infection and in the nucleus and cytoplasm at later stages. As a virulence factor of IAVs and a multifunctional protein, NS1 is involved in a plethora of activities contributing to efficient virus replication and virulence [2]. NS1 contains two important functional domains: an N-terminal RNA-binding domain (RBD), and a C-terminal effector domain (ED), which includes an eukaryotic translation initiation factor 4GI (eIF4GI) binding domain [2]. The RBD domain (aa 1–73) binds several RNAs, including double-stranded RNAs, and poly (A)-containing mRNAs [3, 4]. The C-terminal effector domain (aa 74–230) predominantly mediates interactions with various host–cell proteins to inhibit the cleavage, nuclear–cytoplasmic export, and polyadenylation of host mRNAs; in consequence, this domain prevents the activation of the retinoic acid-inducible gene I-like receptor (RLR) signal transduction pathway and interferon-inducing proteins, which are critical mediators of antiviral innate immune responses [5–7]. The eIF4GI-binding domain requires amino acids 81–113. Previous studies proposed a model where NS1 recruits eIF4GI specifically to the 5’-untranslated region of the viral mRNA, allowing its preferential translation [8, 9].

Scholars have mostly focused on the RBD and ED domains of NS1; however, few studies investigated the eIF4GI-binding domain, which interacts with the host protein eIF4GI [8, 10]. In our previous studies, we demonstrated that recombinant H5N1 virus with complete NS1 eIF4GI-binding domain (rNS1-wt) displayed the highest pathogenicity among other truncated mutant viruses with eIF4GI-binding domains of different lengths. In particular, an NS1 mutant virus with a 30 amino acid deletion (residues 80 to 109) in NS1 (rNS1-SD30) displayed a markedly attenuated replication potential both *in vitro* and *in vivo* and induced the highest levels of IFN-β in MDCK cells [11]. Thus far, limited information is available regarding host factors that affect the differential pathogenicity exhibited by H5N1 viruses with complete or absent eIF4GI-binding domains.

To further elucidate the pathogenic mechanisms affected by the eIF4GI-binding domain of the H5N1 avian influenza virus, we performed 2-dimensional electrophoresis (2-DE) coupled with MS-based quantitative proteomics to systematically characterize differentially expressed (DE) proteins in chicken embryo fibroblasts (CEFs) infected with rNS1-wt and rNS1-SD30 viruses. Our data provides direct insights into the role of NS1 eIF4GI-binding domain in the avian host response to H5N1 avian influenza virus infection, and may help design therapeutic strategies to reduce the high morbidity and mortality caused by IAVs.

## RESULTS

### Replication of NS1 wild-type and NS1 mutant rH5N1 influenza viruses in CEFs

**Figure 1:**
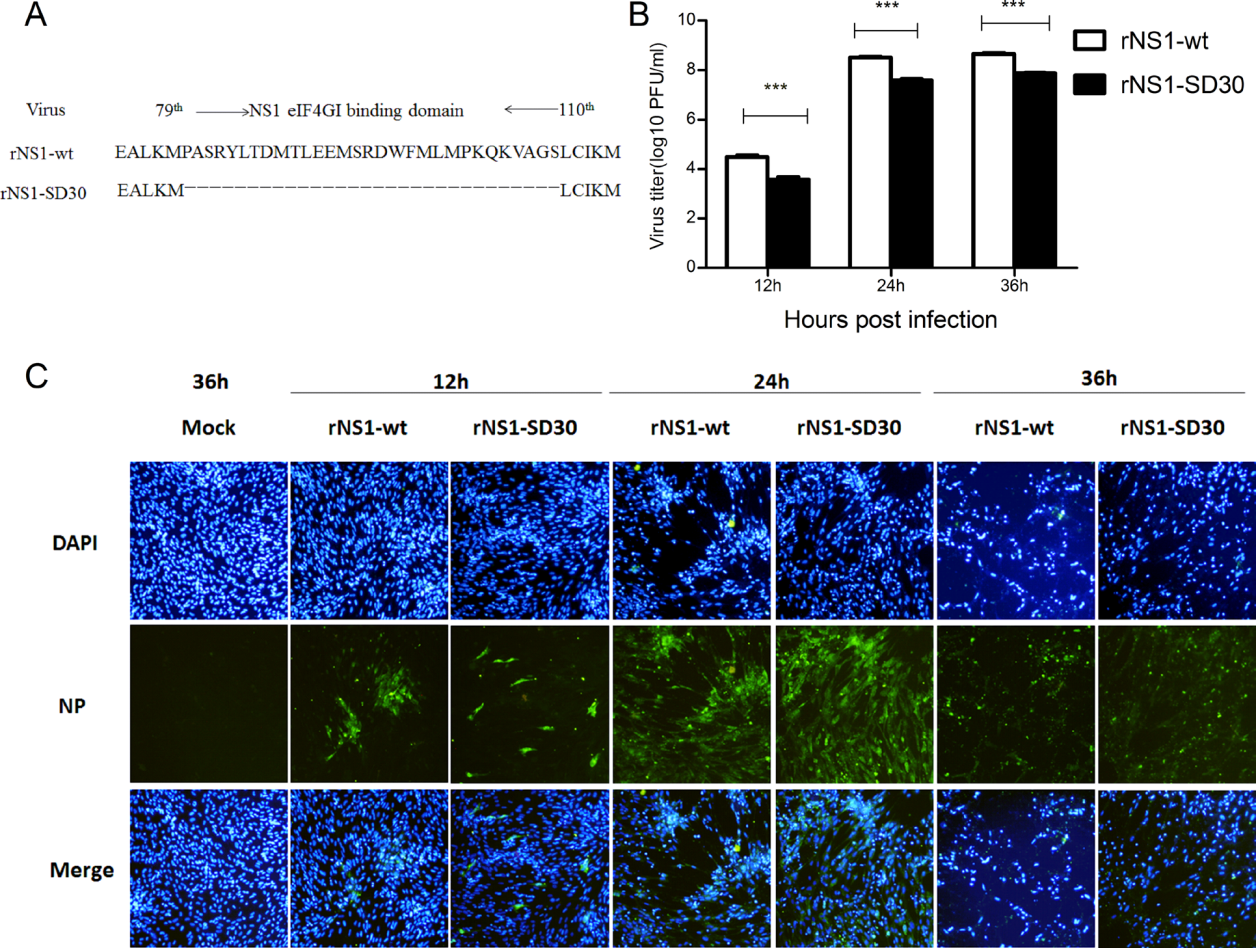
Infection and replication of rNS1-wt and rNS1-SD30 viruses in CEFs (**A**) Schematic diagram of NS gene segments in rNS1-wt and rNS1-SD30 viruses. (**B**) CEFs were infected with rNS1-wt and rNS1-SD30 viruses at 0.01MOI, and supernatants were collected for detecting virus titers at 12, 24, and 36 hpi by standard plaque assay (PFU) in MDCK cells. (**C**) Dynamics of rNS1-wt and rNS1-SD30 proliferation by indirect immunofluorescence (IFA) staining with an anti-NP mAb in infected CEFs at 12, 24, and 36 hpi, respectively, and in mock-infected cells at 36 h as a control. Viral NP proteins are stained green, cell nuclei are stained blue.

We previously showed that rNS1-wt has higher 50% egg infective dose (EID50) and hemagglutination assay (HA) titer, shorter mean death time (MDT) in chicken eggs, and larger plaque size and a 300-fold higher virus titer in MDCK cells than rNS1-SD30. *In vivo* experiments, rNS1-wt (virus titers: 3.6 × 10^8^ PFU/mL) showed the highest virus titer and mortality in chicken and mice, whereas rNS1-SD30 (virus titers: 1.1 × 10^6^ PFU/mL) presented the lowest [11]. Therefore, the rNS1-wt (with complete NS1 eIF4GI binding domain) and the rNS1-SD30 (with deleted NS1 eIF4GI-binding domain) strains were selected for proteomic studies (Figure 1A). For comparison of viral growth kinetics, CEFs were infected with rNS1-wt and rNS1-SD30 viruses at 0.01 multiplicity of infection (MOI), and culture supernatants were collected 12, 24, and 36 h post-infection (hpi) to detect virus titers by plaque assay. Results showed that rNS1-wt virus titers were 10-fold higher than those of rNS1-SD30 at all time points (Figure 1B). Meanwhile, an indirect immunofluorescence assay (IFA) for NP was carried out to verify infection and cytopathic effect (CPE) in CEFs. Both rNS1-wt and rNS1-SD30 viruses effectively replicated in CEFs, and no detectable CPE occurred after 12 h. Whereas CPE was obvious at 24 h, and very severe at 36 h, in CEFs infected with rNS1-wt, CPE in rNS1-SD30-infected cells was only a slight at 24 h and obvious, but less marked than in rNS1-wt infected cells, at 36 h (Figure 1C). Considering the severe CPE caused by the rNS1-wt virus 36 hpi, rNS1-wt- or rNS1-SD30-infected CEFs collected 12, 24, and 36 hpi were selected for the proteomic analyses to identify DE proteins.

### 2-DE profiles and identification of differentially expressed proteins

**Figure 2:**
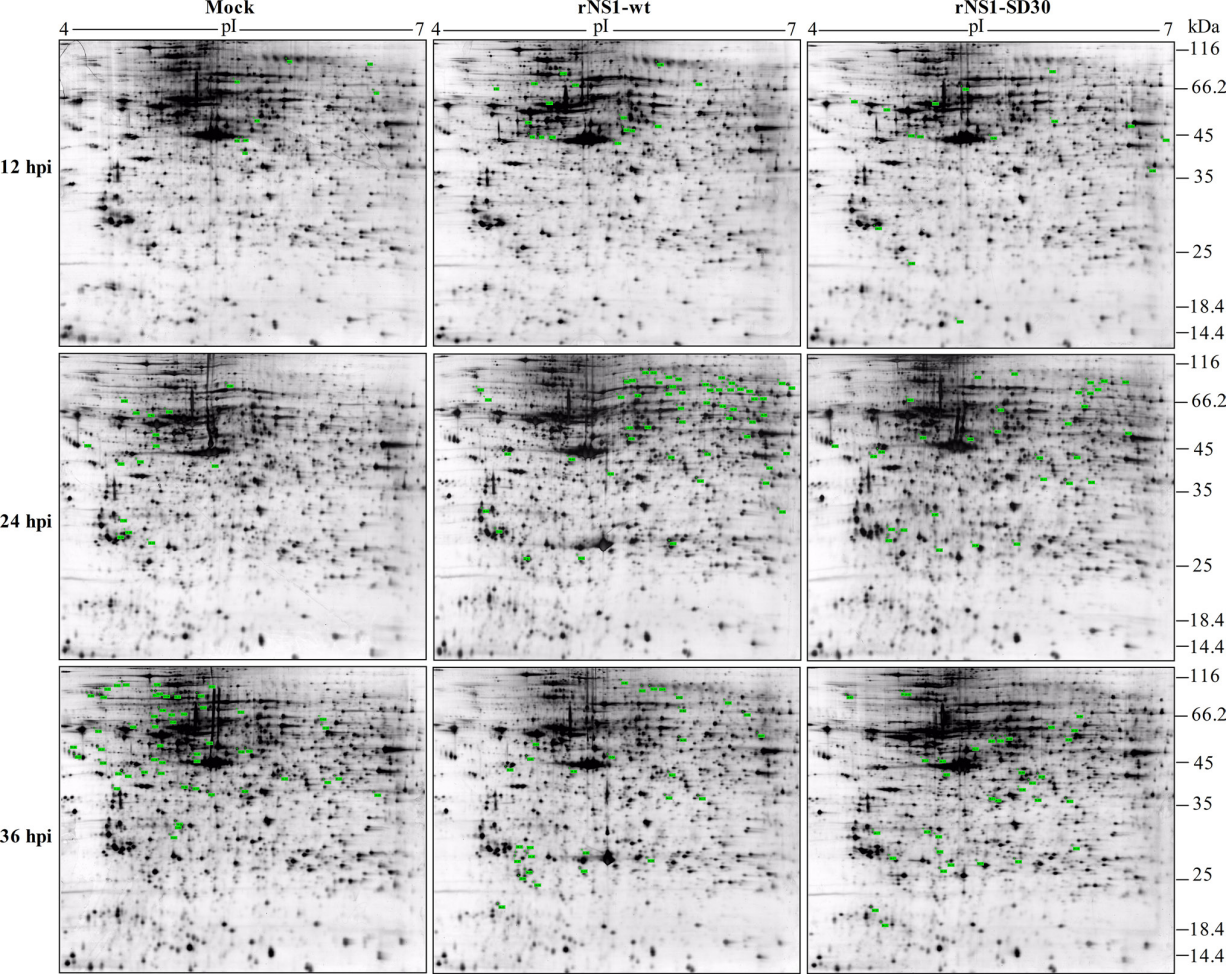
Representative 2-D gel maps of DE protein spots in infected and uninfected CEFs The DE proteins detected are marked by green dots. Detailed information is listed in a to g refer to the corresponding footnotes in Table 1, Supplementary Table 3 and 4 with corresponding numbers. The inoculations are indicated at the top and the time points for CEF collection are shown on the left. Representative images generated from samples in each group are shown.

Proteins extracted at 12, 24, and 36 hpi from CEFs infected with rNS1-wt and rNS1-SD30 viruses and from uninfected (PBS-treated) control CEFs were prepared and loaded on to 2-DE gels for separation. To compensate for gel electrophoresis variability, we ran three replicate gels for each time point. Based on image analysis by PDQuest software, pairwise comparisons of relative protein abundance were performed among the three groups (rNS1-wt versus control, rNS1-SD30 versus control, and rNS1-wt versus rNS1-SD30). Visual analysis of gels indicated a total of 133 DE protein spots with a minimum of 1.5-fold change across a pI range of 4–7 among the three groups from each time point (Figure 2). The DE protein spots are marked with numbers in the images.

**Figure 3:**
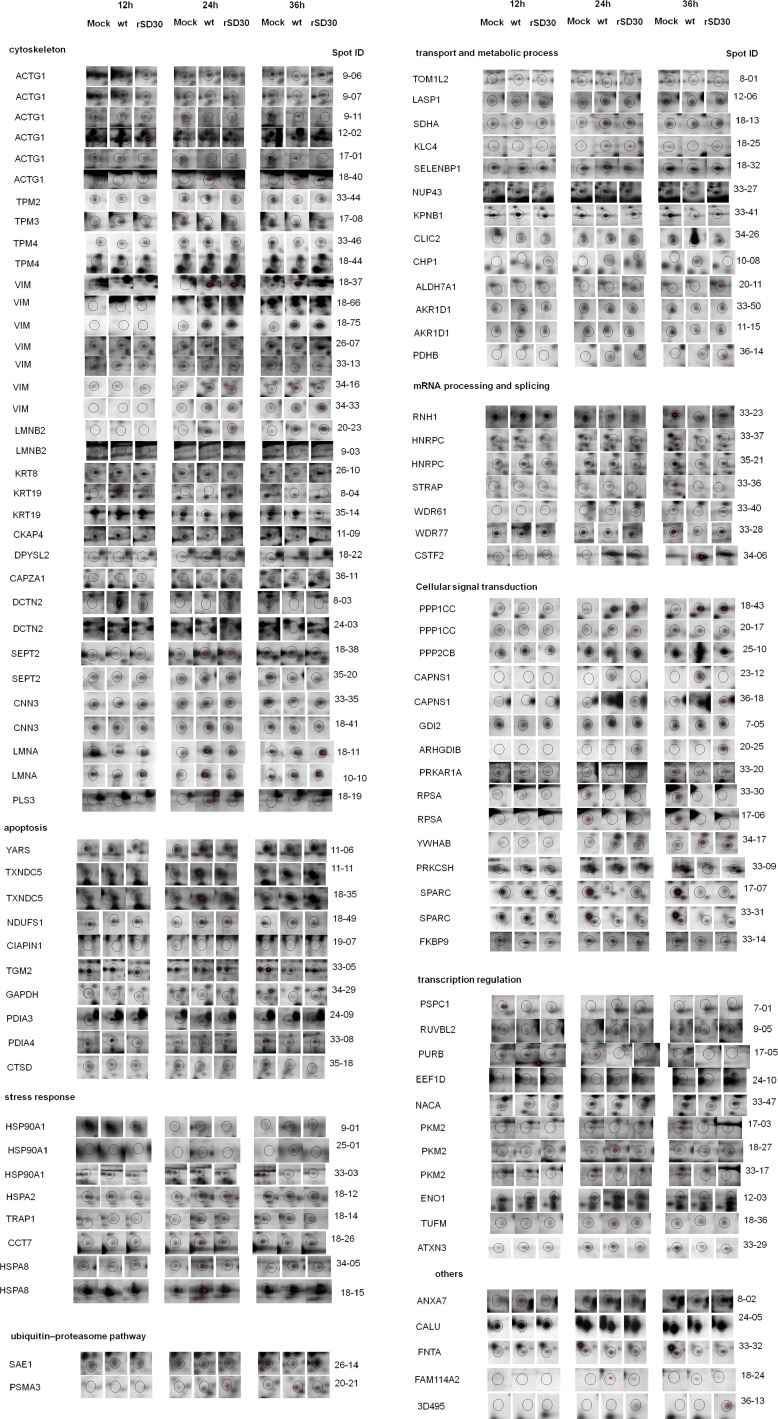
Enlarged 2-DE profiles of DE protein spots in rNS1-wt- and rNS1-SD30-infected CEFs Circled dots indicate the DE protein spots. MOCK indicates regulated protein spots in uninfected CEFs. wt and SD30 indicate protein spots of CEFs infected with rNS1-wt and rNS1-SD30 viruses, respectively.

The DE protein spots were excised and subjected to MALDI-TOF MS/MS analysis. Every protein spot was identified by a minimum of two peptides. In total, 23, 50, and 60 DE protein spots were identified at 12, 24, and 36 hpi respectively (Table 1, Supplementary Table 3, and Supplementary Table 4). At 12 hpi, rNS1-wt-infected CEFs presented 4 upregulated and 4 downregulated protein spots, whereas rNS1-SD30-infected CEFs presented 4 upregulated and 7 downregulated protein spots relative to uninfected control cells. At 24 hpi, rNS1-wt-infected CEFs had 28 upregulated and 7 downregulated protein spots, whereas rNS1-SD30-infected CEFs had 16 upregulated and 5 downregulated protein spots. At 36 hpi, there were 16 upregulated and 25 downregulated protein spots in rNS1-wt-infected CEFs, and 11 upregulated and 19 downregulated protein spots in rNS1-SD30-infected CEFs. Comparison between rNS1-wt and rNS1-SD30 CEFs revealed 7 upregulated and 5 downregulated protein spots at 12 hpi, 5 upregulated and 5 downregulated protein spots at 24 hpi, and 8 upregulated and 12 downregulated protein spots at 36 hpi. Several proteins, such as ACTG1, vimentin, and CAPNS1, which were identified from more than two different spots on the gel, matched the same protein in the NCBI nr database; moreover, the spots were clearly separated in the gels, suggesting that the proteins may undergo post-translational modification (PTM) or certain types of cleavages. The majority of the DE protein spots are illustrated in enlarged form according to their functional classification (Figure 3).

**Table 1:** Pairwise comparison of differentially expressed proteins in CEF at 12 hours post-inoculation with the H5N1 viruses rNS1-wt or rNS1-SD30

Spot ID^a^	Protein name (Abbreviation)	Accession No^b^	Differentially expressed proteins identified in CEF between groups						Protein score^d^	Matched peptide^e^
			rNS1-wt and Mock		rNS1-SD30 and Mock		rNS1-wt and rNS1-SD30			
			*P*-value	Ratio^c^	*P*-value	Ratio	*P*-value	Ratio		
7-01	paraspeckle component 1 (PSPC1)	gi|71895115	2.9e-19	-2.14	2.9e-19	-100^f^	- g	-	247	11
7-03	T-complex protein 1 subunit zeta(CCT6A)	gi|57525300	1.1e-08	-2.88	-	-	-	-	141	7
7-05	Rab GDP dissociation inhibitor beta (GDI2)	gi|45384364	2.3e-05	-1.85	-	-	2.3e-05	-1.64	108	7
7-06	alpha-2- macroglobulin(A2M)	gi|157954061	0.033	-2.04	-	-	0.033	-2.08	76	2
8-01	TOM1-like protein2(TOM1L2)	gi|118097857	2.9e-15	100	-	-	-	-	207	10
8-02	annexin A7 (ANXA7)	gi|50749404	2.3e-13	100	-	-	-	-	188	15
8-03	Dynactin subunit 2 (DCTN2)	gi|45382201	1.4e-14	100	1.4e-14	9.13	1.4e-14	10.95	200	6
8-04	keratin, type I cytoskeletal 19(KRT19)	gi|45384356	2.3e-26	8.75	2.3e-26	6.02	-	-	318	16
9-01	heat shock protein HSP 90-alpha (HSP90AA1)	gi|157954047	-	-	7.2e-18	-2.98	7.2e-18	3.34	233	8
9-03	lamin-B2(LMNB2)	gi|45384202	-	-	9.1e-11	-2.0	-	-	162	13
9-05	RUVB-like 2(RUVBL2)	gi|148230609	-	-	7.2e-05	-2.2	-	-	103	7
9-06	actin, cytoplasmic type 5(ACTG1)	gi|56119084	-	-	3.6e-21	-2.04	-	-	266	7
9-07	actin, cytoplasmic type 5(ACTG1)	gi|56119084	-	-	7.2e-26	-2.21	7.2e-26	1.8	313	8
9-11	actin, cytoplasmic type 5(ACTG1)	gi|56119084	-	-	4.5e-17	-1.58	-	-	225	6
10-08	calcium-binding protein p22(CHP1)	gi|56118996	-	-	3.6e-21	100	-	-	266	5
10-10	lamin-A (LMNA)	gi|45384214	-		2.9e-25	1.51	-	-	307	22
11-06	tyrosyl-tRNA synthetase, cytoplasmic(YARS)	gi|57530465	-	-	-	-	4.5e-16	1.66	215	
11-09	cytoskeleton-associated protein 4(CKAP4)	gi|118082813	-	-	-	-	5.8e-15	1.65	204	8
11-11	thioredoxin domain-containing protein 5(TXNDC5)	gi|57530789	-	-	-	-	1.1e-22	100	281	6
11-15	Aldo-keto reductase family 1 member D19(AKR1D1)	gi|118082901	-	-	-	-	0.00011	1.54	101	6
12-02	actin, cytoplasmic type 5(ACTG1)	gi|56119084	-	-	-	-	1.1e-07	-2.46	131	5
12-03	alpha-enolase(ENO1)	gi|46048768	-	-	-	-	9.1e-18	-1.75	232	8
12-06	LIM and SH3 domain protein 1(LASP1)	gi|293601661	-	-	-	-	1.4e-11	-2.54	170	6

^a^Spot ID represents the number on the representative gel in Figure 2.

^b^Accession number is the MASCOT result of a MALDI-TOF/TOF search of the NCBInr database.

^c^Ratio stands for the average protein abundance ratio for different groups. A positive value indicates that protein expression was up-regulated in the first group; a negative value indicates that protein expression was down-regulated in the first group.

^d^MASCOT score is−10* log(p), where p is the probability that the observed match is a random event. Based on the NCBInr database using the MASCOT searching program as MS/MS data. Scores greater than 74 are significant (p < 0.05).

^e^The number of peptides identified by using MALDI-TOF/TOF is given by MASCOT.

^f^The ratio of 100 indicates that the protein spots were newly induced (100) or absent (-100) in one virus-infected CEF relative to the other virus infected CEF or mock CEF.

^g^The symbol “-” means the fold ratios of spots are not significant.

### Bioinformatics analyses of differentially expressed proteins

To obtain functional insights into the biological processes affected by the DE proteins identified after comparing rNS1-wt- and rNS1-SD30- infected CEFs, the UniProt knowledgebase (Swiss-Prot/TrEMBL) and the Gene Ontology database were examined. The identified cellular proteins were functionally classified into several categories, which included cytoskeleton, apoptotic and stress response, transcription regulation, transport and metabolic process, mRNA processing and splicing, and cellular signal transduction. Detailed functional information for the identified DE proteins is shown in Supplementary Table 1.

**Figure 4:**
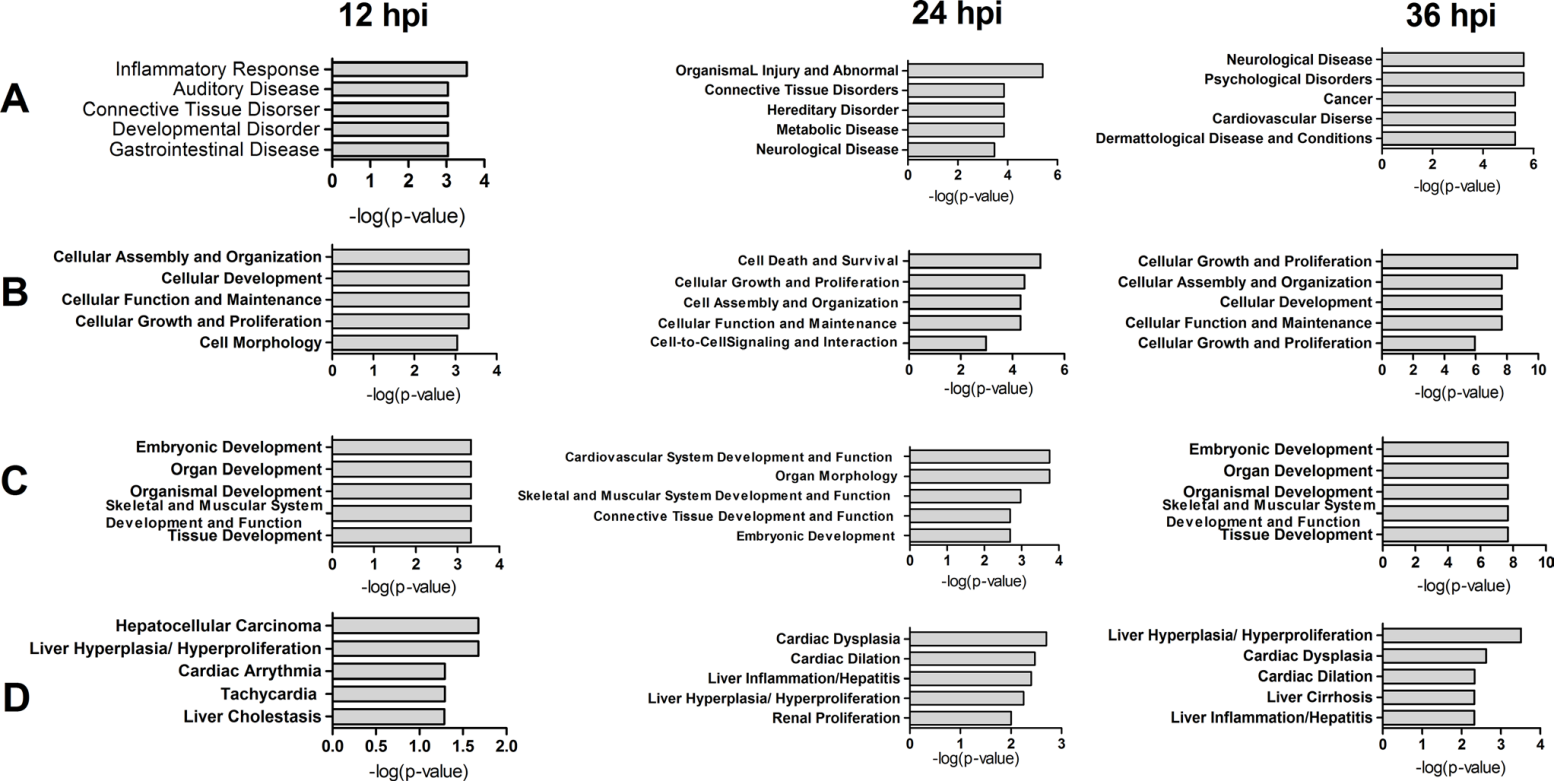
Top 5 diseases and bio functions of DE proteins identified by comparing rNS1-wt and rNS1-SD30 infections at 12, 24 and 36 hpi (**A**) Diseases and Disorders; (**B**) Molecular and Cellular Functions; (**C**) Physiological System Development and Functions; and (**D**) Toxicity Functions.

To analyze function classifications and interacting networks in which the DE proteins are involved, the gi numbers of the identified proteins were imported into the Ingenuity Pathways Analysis (IPA) tool. Function classification analysis by IPA divided these proteins into four distinctive functional sets: diseases and disorders, molecular and cellular functions, physiological system development and functions, and toxicity functions. The top five statistically significant levels are depicted in Figure 4. These DE proteins were involved in many biological functions including cellular assembly and organization, cell death and survival, cellular growth and proliferation, cellular development, cellular function and maintenance, cell morphology, and cell-to-cell signaling and interaction.

**Figure 5:**
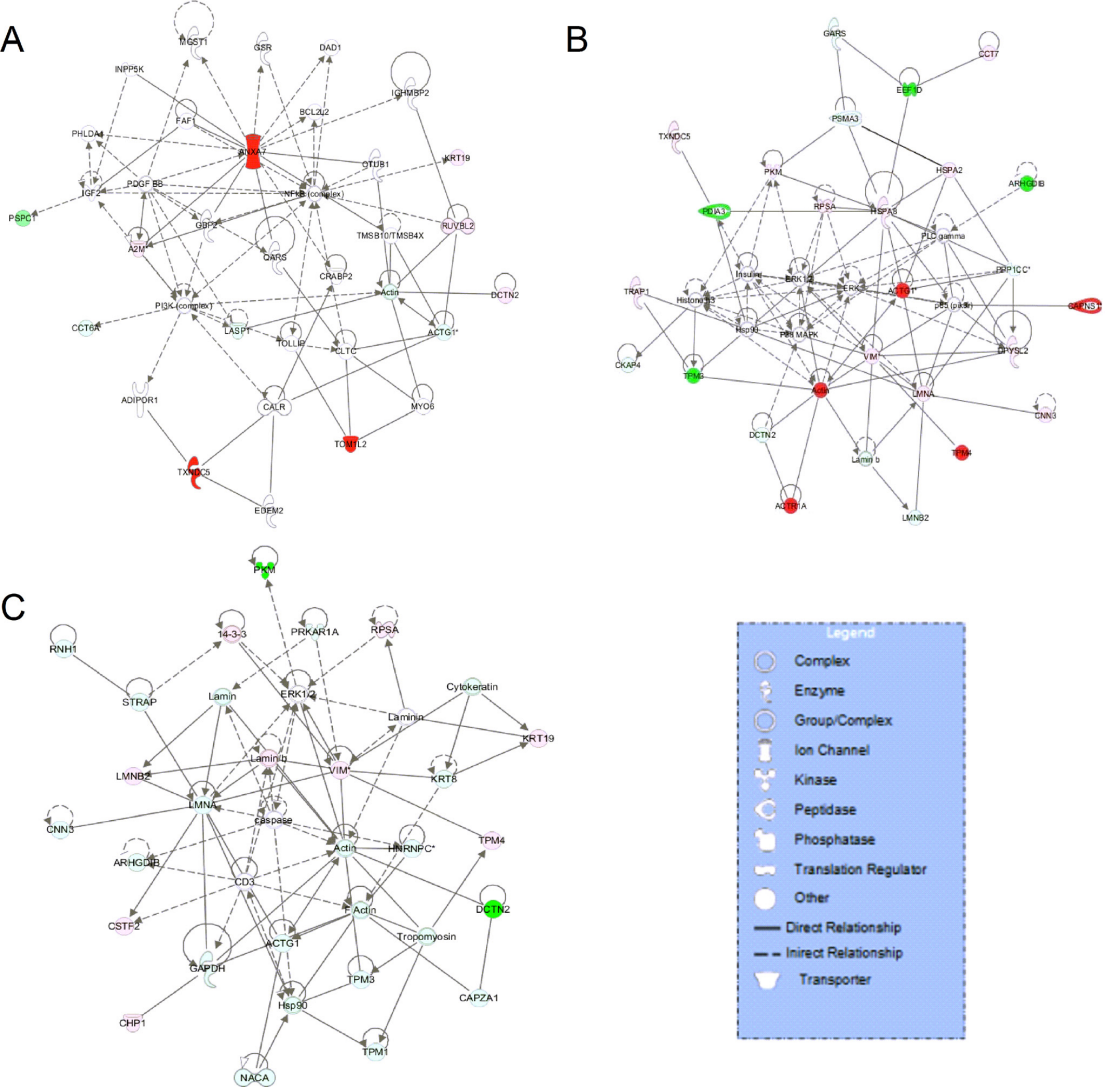
Comparative network pathway analysis DE proteins Highest-score network at 12 h (**A**), 24 h (**B**) and 36 h (**C**). Red, upregulated proteins; green, downregulated proteins; white, proteins known to be in the networks but not identified in our study. Color intensities indicates the degree of the change in protein expression levels. The shapes of the symbols are indicative of the molecular class. Lines connecting molecules indicate molecular relationships. Solid lines: direct known interactions; dashed lines: suspected or indirect interactions; arrows: specific molecular relationships and directionality of the interaction.

According to the analysis of interacting networks by IPA, in total, 2, 2, and 3 specific functional networks were mapped at 12, 24, and 36 hpi, respectively (Figure 5 and Supplementary Figure 1). At 12 hpi, DE proteins detected upon comparison of rNS1-wt- and rNS1-SD30-infected CEFs that were mainly involved in cellular function and maintenance, cell-to-cell signaling and interaction, and inflammatory response were linked to the NF-κB and PI3K pathways, which are important for efficient replication of the influenza virus (Figure 5A). At 24 hpi, the DE proteins primarily involved in connective tissue disorders, hereditary disorder, and metabolic disease were linked to the p38 MAPK and ERK 1/2 pathways (Figure 5B). At 36 hpi, the DE proteins involved in cellular assembly and organization, cellular development, and cellular function and maintenance were linked to the ERK 1/2 and caspase pathways (Figure 5C). The referred pathways are all canonical and support viral propagation.

### Validation of the differentially expressed proteins by western blot

**Figure 6:**
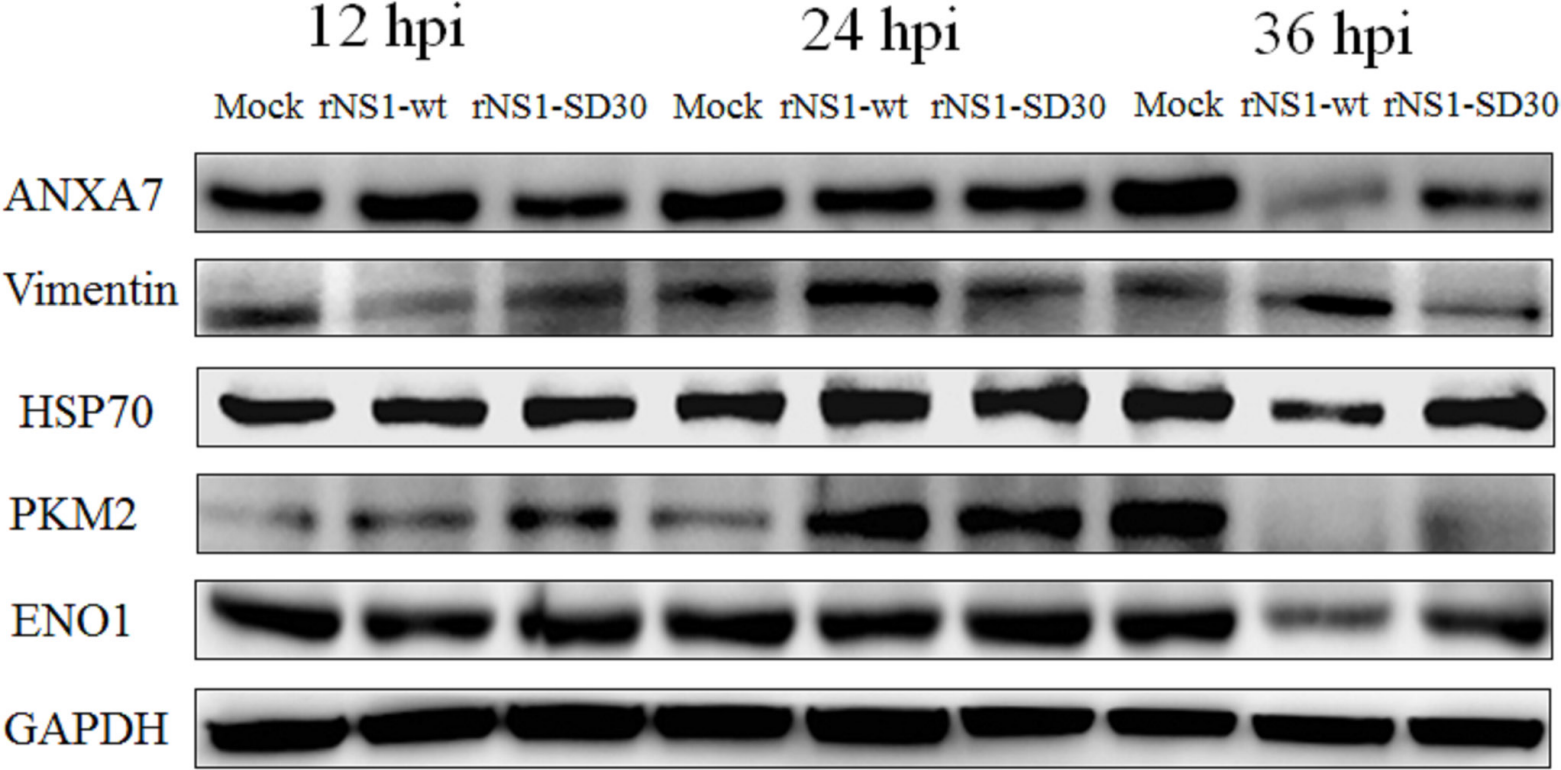
Western blots of representative proteins Proteins were detected from individual samples of rNS1-wt-, rNS1-SD30-, and mock-infected CEFs collected at 12, 24, and 36 hpi.

To validate the results of our 2-DE/MALDI-TOF MS/MS data, we selected six DE proteins detected at all three time points (ANXA7, vimentin, HSP70, PKM2, ENO1, and GAPDH) for western blot analysis. GAPDH was used as endogenous control, as it showed no significant expression changes between treatments. At 12 hpi, the amount of ANXA7 in rNS1-wt infected CEFs was increased relative to control cells, which showed similar expression levels to those of rNS1-SD30-infected CEFs. At 24 hpi, in contrast, ANXA7 expression was similar between groups. At 36 h, ANXA7 levels were markedly decreased in both rNS1-wt- and rNS1-SD30-infected CEFs. At all time points, vimentin expression was downregulated in CEFs infected with both virus strains, except for rNS1-wt-infected CEFs that showed vimentin upregulation at 36 h (Figure 6). We speculate that the discrepancy between these data and those from 2-DE gels, in which vimentin levels were consistently upregulated or downregulated at all three time points in infected versus uninfected cells, may be related to PTM. Meanwhile, western blot levels of other proteins, such as HSP70, PKM2, and ENO1, were consistent with proteomic analyses.

### Identification of host proteins contributing to rNS1-wt and rNS1-SD30 viruses propagation

**Figure 7:**
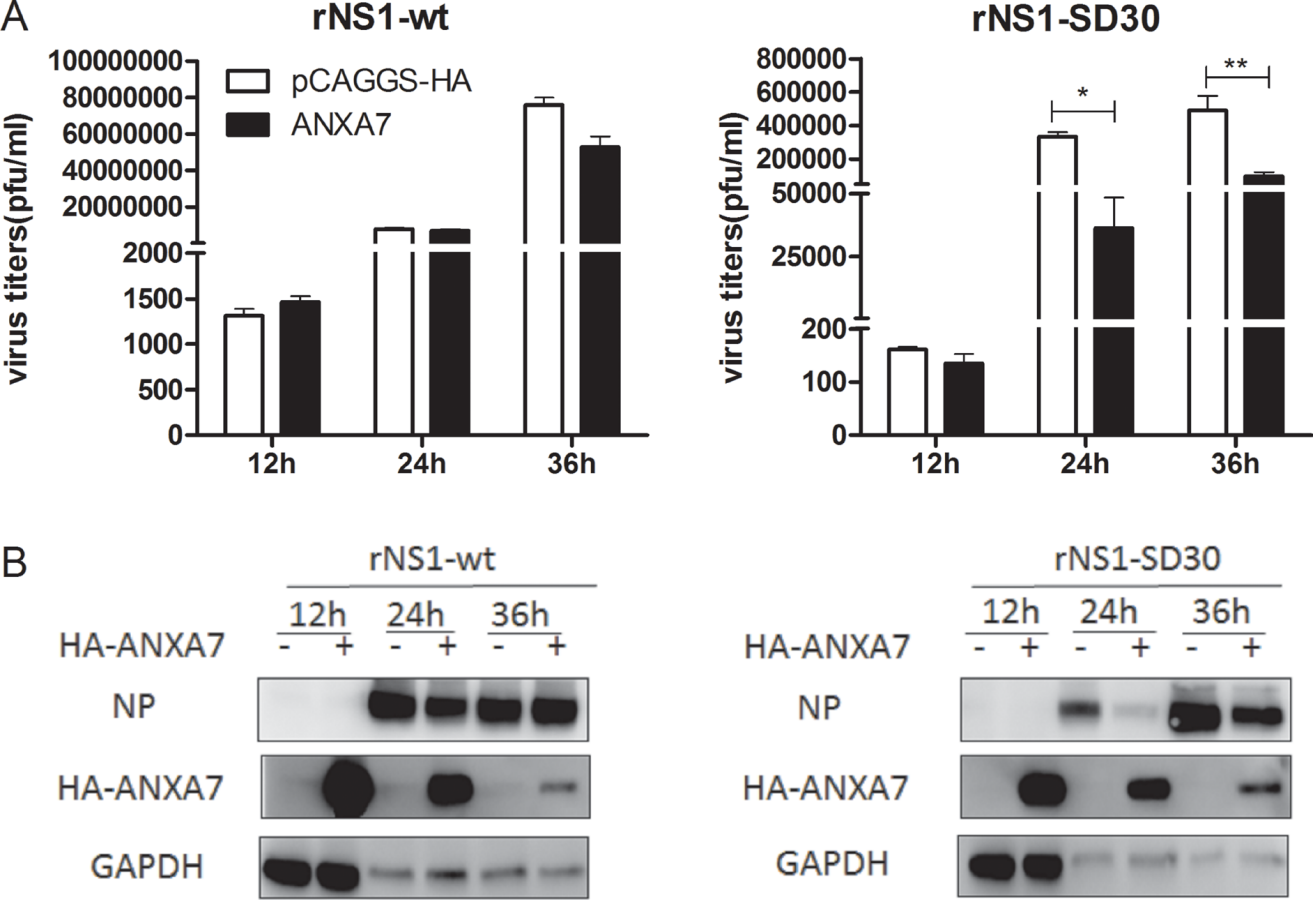
Overexpression of ANXA7 inhibits rNS1-SD30 virus replication (**A**) DF-1 cells were transfected with 1µg HA-ANXA7 or control plasmid (pCAGGS-HA) for 24 h, and subsequently infected with rNS1-wt and rNS1-SD30 viruses at 0.01 MOI. Supernatants were collected at 12, 24, and 36 hpi, and assessed for viral titers using plaque assays on MDCK cells. (**B**) DF-1 cells were transfected with 2 µg HA-ANXA7 or control plasmid (pCAGGS-HA) for 24 h, and infected with rNS1-wt and rNS1-SD30 viruses at 0.01 MOI. Cell lysates collected at 12, 24, and 36 hpi were subjected to western blotting to assess NP and ANXA7 protein levels with NP and HA tag antibodies.

To investigate the effect of the DE proteins on rNS1-wt and rNS1-SD30 virus propagation, 26 plasmids coding DE proteins, or empty control plasmids (Supplementary Table 2) were transfected into DF-1 cells. After infection with rNS1-wt or rNS1-SD30 viruses at 0.01 MOI, viral titers were determined in cell supernatants at 12, 24 and 36 hpi. Results showed that at 12 hpi, rNS1-SD30 virus titers were increased by GARS and decreased by RPSA and CAPZA1. At 24 hpi, 3D495, PKM2, and YWHAB increased rNS1-wt virus titers, whereas GARS and IMMT increased, and ANXA7 substantially decreased, rNS1-SD30 titers. At 36 hpi, CAPNS1, CHP, EEF1D, PDIA3, and TXNDC5 increased, while PURB suppressed, rNS1-wt titers, while LASP1, PDIA3, and TXNDC5 augmented rNS1-SD30 viral titers (Supplementary Figure 2). Interestingly, ANXA7 overexpression significantly inhibited rNS1-SD30 replication compared with control, but exerted no effect on rNS1-wt (Figure 7A). Indeed, compared with control cells, a greater than 10-fold reduction in infectious virions was found in the supernatant of ANXA7-overexpressing cells infected with rNS1-SD30 virus at 24 and 36 hpi. Viral NP protein was next detected by western blot. Consistent with virus titer results, NP expression was markedly suppressed after ANXA7 overexpression only in cells infected with rNS1-SD30 (Figure 7B). These data suggest that ANXA7 is particularly relevant for rNS1-SD30 virus replication in DF-1 cells.

### Impact of DE protein overexpression on chMDA5-induced IFN-β production

**Figure 8:**
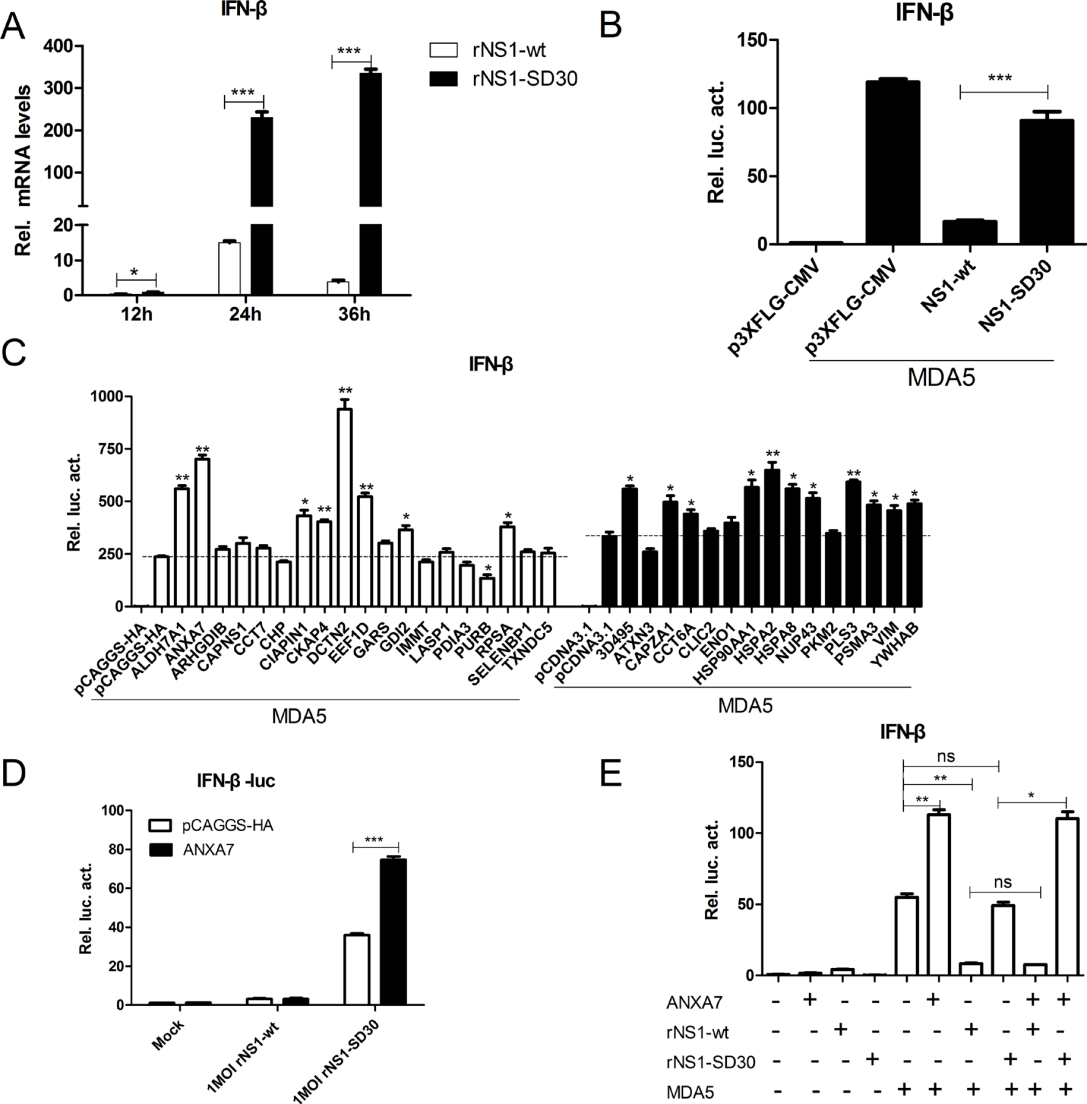
ANXA7 promotes the IFN-β response (**A**) mRNA expression levels in CEFs infected by rNS1-wt and rNS1-SD30 viruses. Uninfected and rNS1-wt- and rNS1-SD30- infected CEFs (0.01 MOI) were collected at 12, 24, and 36 hpi. Total RNA extracted from mock and virus-infected cells was used to detect chicken IFN-β mRNA by qRT-PCR. (**B**) NS1-wt inhibits IFN-β promoter activity induced by chMDA5. DF-1 cells were transfected with chMDA5 (100 ng) or empty vector (100 ng) and the indicated expression plasmids (200 ng) together with the pGL3-chIFN-β reporter plasmid (100 ng) and the pRL-TK vector (10 ng) for 24 h. Cells were lysed prior to luciferase assays; expression results reflect comparison to luciferase activity in control cells. (**C**) Effect of proteins overexpress on chMDA5 induced IFN-β response. DF1 cells were transfected with the indicated expression plasmids (200 ng) for 24 h, and subjected to luciferase assays as described in (B). (**D**) ANXA7 promotes IFN-β promoter activity induced by rNS1-SD30 virus. DF-1 cells were transfected with the indicated plasmids for 24 h, and subsequently infected with 1 MOI rNS1-wt or rNS1-SD30 virus or left untreated for 24 h. Luciferase assay was performed as described in (B).

Since the NS1 protein of the influenza virus is known to suppress IFN-β production, we used qRT-PCR to examine the ability of rNS1-wt and rNS1-SD30 viruses to induce IFN-β in CEFs. Results showed that the rNS1-SD30 virus induced significantly higher (3-, 15-, and 100-fold at 12, 24, and 36 h, respectively) IFN-β mRNA levels than the rNS1-wt virus (Figure 8A). Then, NS1-wt and NS1-SD30 constructs were overexpressed in DF-1 cells to investigate their effect on chMDA5-induced IFN-β promoter activity. Data showed that NS1-wt significantly suppressed IFN-β promoter activity compared with empty plasmid; in contrast, no apparent inhibition of IFN-β promoter activity was observed when NS1-SD30 was overexpressed (Figure 8B).

Next, the effects of 26 overexpression plasmids on chMDA5-induced IFN-β promoter activity were studied. Results indicated that several DE proteins were able to modulate IFN-β promoter activity, which was strongly enhanced by ALDH7A1, ANXA7, and DCTN2, slightly enhanced by EEF1D, GDI2, RPSA, 3D495, CAPZA1, CCT6A, HSPA8, PLS3, PSMA3, VIM, and YWHAB, and decreased by PURB overexpression (Figure 8C). Based on the ability of ANXA7 to inhibit rNS1-SD30 virus propagation, we next assessed the potential role of ANX7 on the IFN-β response of DF-1 cells infected with rNS1-wt or rNS1-SD30 viruses. ANXA7 overexpression increased IFN-β promoter activity in rNS1-SD30-infected cells, while no effect was detected for rNS1-wt (Figure 8D). Similar results were observed during evaluation of chMDA5-induced IFN-β promoter activity after co-transfection of DF-1 cells with ANXA7 and NS1-wt or NS1-SD30. Namely, ANXA7 overexpression enhanced chMDA5-induced IFN-β promoter activity in cells co-transfected with NS1-SD30, but inhibited it upon co-transfection with NS1-wt (Figure 8E). The fact that stimulation of IFN-β promoter activity by ANXA7 can be counteracted by NS1-wt indicates that ANXA7 may be a key host factor in type-I IFN signaling pathway induction by H5N1.

## DISCUSSION

In our previous study, a recombinant H5N1 virus lacking the eIF4GI binding domain of the NS1 protein (rNS1-SD30) displayed a highly attenuated phenotype compared with the rNS1-wt virus possessing an intact eIF4GI-binding domain [11]. This domain permits direct interaction of the influenza virus NS1 protein with the human host factor eIF4GI both *in vitro* and *in vivo*, thus improving virus translation [8]. Deletion of the eIF4GI-binding domain affects the binding ability of NS1 protein to eIF4GI, resulting in an impairment of the initial rate of viral mRNA translation. However, the molecular mechanisms underlying different avian host responses to infections with H5N1 influenza virus with complete or absent NS1 eIF4GI-binding domains remain unclear. The goal of this proteomic study was to assess, using both primary chicken embryo fibroblasts (CEFs) and immortalized (DF-1 cells), the pathogenic mechanisms mediating differential host cell responses to rNS1-wt and rNS1-SD30 viruses, which are genetically similar but markedly different in their pathogenic characteristics. We identified 81 proteins that were differentially expressed at different time points after infection. Bioinformatic analysis indicated that these host proteins are involved in cytoskeleton, apoptosis, stress response, transcription regulation, transport and metabolic process, mRNA processing and splicing, and cellular signal transduction.

In mammals, the type I interferon (IFN) response represents the first line of defense against influenza virus infection. Our data confirms that after infection with this pathogen avian cells such as CEFs and DF-1 cells also produce IFN [12]. We show here that the rNS1-SD30 virus induces higher IFN-β mRNA expression levels than the rNS1-wt virus in CEFs. This is in agreement with a study that reported that wild-type IAVs are poor inducers of type I IFN [13]. The RLRs family plays essential roles in the sensing of RNA viruses; the most extensively characterized RLR to date is RIG-I, whose activation, leading to IFN induction, is suppressed by the NS1 protein of AIVs [14]. However, unlike mammals and some birds like ducks, chickens lack RIG-I, therefore chMDA5 acts as the pattern recognition receptor responsible for sensing IAV infections in chicken cell lines [15, 16]. We found that the IFN-β promoter activity induced by chMDA5 was significantly suppressed by overexpression of NS1-wt, but not NS1-SD30, in chicken DF-1 cells. Although IFN effector molecules and antiviral IFN-stimulated genes were not identified in our proteomic results, we detected several proteins involved in the immune response. ACTG1 (actin, cytoplasmic type 5) was downregulated by rNS1-SD30 infection at 12 h, and upregulated upon infection with both viruses at 24 and 36 h compared with uninfected controls. Previous studies reported that in response to IAV infection in macrophages, actin can translocate from cytoplasm to mitochondria along with several components of the RIG-I/MAVS signaling pathway, including RIG-I, TRADD, TRIM25, and IKKε, and then lead to activation of an antiviral response [17]. PPP1CC (also known as PP1γ) was upregulated in cells infected with either virus at 24 and 36 h. Past research reported that PP1α and PP1γ are recruited to RIG-I and MDA5 upon virus infection or viral RNA binding, leading to the dephosphorylation of their N-terminal CARDs and consequent activation of the RLR signaling pathway [18]. Also, PP1 was reported to repress immune signaling by negatively regulating TLR- and RLR-triggered IFN-β production by dephosphorylating IRF3 and targeting TGF-β–activated kinase 1 (TAK1) serine 412 phosphorylation [19, 20]. In addition, PP1 is recruited to dephosphorylate IKK, negatively regulating NF- kB signaling [21]. PPP2CB (also known as PP2Cβ and PPM1B) was upregulated in rNS1-wt-infected cells at 36 h. PPM1B performs an important role in the termination of TNF α-mediated NF-κB activation through dephosphorylation and inactivation of IKKβ [22], and also negatively modulates type I IFN signaling by dephosphorylating TBK1 at serine 172 [23]. Whether these proteins perform different roles in regulating the type-I IFN response to rNS1-wt and rNS1-SD30 virus infection in chicken cells requires further investigation.

We also identified several previously unreported proteins that may modify IFNβ promoter activity induced by chMDA5. For example, ALDH7A1 and DCTN2, which were upregulated in rNS1-SD30-infected cells, strongly enhanced IFN-β promoter activity induced by chMDA5. For instance, ANXA7 was upregulated by rNS1-wt at 12 hpi. We showed that ANXA7 overexpression promotes IFN-β promoter activity induced by both rNS1-SD30 virus and chMDA5, and significantly suppresses virus titers and NP protein expression in cells infected with rNS1-SD30, but not with rNS1-wt. Thus, although further research is warranted, the lower pathogenicity of the rNS1-SD30 virus may be related to the activity of ANXA7, coupled perhaps to that of other immune regulation proteins.

Viruses use the host’s actin and microtubule transport systems in several steps of their life cycle, i.e during attachment, internalization, endocytosis, nuclear targeting, transcription, replication, transport of progeny subviral particles, assembly, exocytosis, and cell-to-cell spread [24]. Certain viral proteins, such as actin-binding proteins, can interact with the cytoskeletal transport machinery and induce rearrangements of cytoskeletal filaments for use as tracks, or push aside filaments that function as barriers [25]. In the present study, a variety of proteins associated with cytoskeletal components such as microfilaments (ACTG1), intermediate filaments (KRT8, KRT19, vimentin, LMNA, and LMNB2), actin-binding proteins (TPM2, TPM3 TPM4, CKAP4, and PLS3), motor proteins (DCTN2 and KLC4), and microfilament-associated proteins (SEPT2, CAPZA1, and CNN3) were identified as differentially expressed at various time points. However, the microtubule protein, β-tubulin, which reportedly interacts with the influenza virus NS1 protein [26], was not found to be differentially regulated by viral infection. Evidence suggests that a fraction of the influenza RNPs and virus matrix M1 protein in infected cells is associated with microfilaments [27], and NP is recognized as an F-actin binding protein [28].

Through its important role in vesicular membrane trafficking and acidification of endosomes, vimentin is required for the release of the influenza genome from late endosomes and for trafficking of virus-containing endosomes toward the nucleus [29]. As numerous DE cytoskeleton-associated proteins were detected when comparing rNS1-wt- and rNS1-SD30-infected chicken cells, further research is needed to assess the interactions with the host cytoskeletal network that appear to be required by each viral strain.

Apoptosis is an important strategy used by host cells to limit virus replication, and serves as an innate defense mechanism against viral infections as well. However, viruses have evolved various strategies to delay apoptosis [30]. Previous studies showed that influenza virus infection causes apoptosis of cells both *in vivo* and *in vitro*, with the viral proteins neuraminidase (NA), NS1, and PB1-F2 playing a role [31–33]. Compared with H1N1, H5N1-infected macrophages show delayed apoptosis onset and contribute to the pathogenesis of H5N1 disease in humans [34]. In this research, multiple apoptosis and stress response-associated proteins, including TXNDC5 (also known as endoplasmic reticulum resident protein, ERP46), PDIA3 (also known as ERp57 and ERp60), and PDIA4 (also known as ERp70 and ERp72) were found to be differently expressed in rNS1-wt and rNS1-SD30-infected CEFs. Members of the protein disulfide isomerase (PDI) family are mainly localized to the endoplasmic reticulum (ER) and function as enzymatic chaperones intervening in the reconstruction of misfolded proteins [35]. Previous studies showed that several PDI variants prevent apoptotic cell death associated with ER stress and protein misfolding in various *in vivo* and *in vitro* models. For example, TXNDC5 upregulation decreases the apoptosis rate in gastric cells [36]. In astrocytes, hypoxia or transient forebrain ischemia causes PDI upregulation, which protects cells against apoptotic death [37]. On the other hand, reduced PDIA3 expression has been reported to increase the apoptotic response to fenretinide in cancer cells [38], and to attenuate the IAV burden while reducing subsequent caspase-12 activation and apoptosis in epithelial cells [39]. We also show that NDUFS1 was upregulated in both rNS1-wt- and rNS1-SD30-infected cells. NDUFS1, the 75 kDa subunit of the respiratory complex I, is a critical caspase substrate that is cleaved during apoptosis, leading to mitochondrial demise [40]. Cathepsin D (CTSD), found here to be downregulated in rNS1-SD30-infected cells, is an important mediator of apoptosis induced through the lysosomal pathway [41]. While apparently involved in the intrinsic apoptosis pathway of activated human T lymphocytes [42], CTSD may also influence resolution of inflammation by inducing apoptosis in neutrophils [43]. The downregulation of apoptosis-associated proteins in CEFs infected by rNS1-SD30 may contribute to its lower pathogenicity by decreasing the apoptosis rate, thus inhibiting viral propagation.

Heat shock proteins (HSPs) are synthesized in response to a wide variety of stressful stimulants, including viral infections, and render the host cells resistant to further and more severe stress [44]. HSPs interact with viral components at different stages of the viral life cycle and may participate in the control of virus replication and morphogenesis [45]. For example, HSP90, an abundant house-keeping protein that is essential for viability in eukaryotic cells, interacts with the PB1 and PB2 subunits of the influenza RNA polymerase, suggesting a role as a molecular chaperone during assembly or nuclear transport of these subunits prior to the formation of a mature ternary polymerase complex [46, 47]. HSP90AA1 was downregulated in rNS1-SD30-infected cells at 12 hpi, upregulated in rNS1-wt -infected cells at 24 hpi, and both up-regulated and down-regulated in cells infected with both viruses at 36 hpi. These variations indicate that HSP90AA1 may be modified by other proteins. HSP70 is an influenza M1 protein and non-structural protein 2- (NS2) binding factor that plays a role in the nuclear transport of vRNP complexes [48, 49]. The HSP70 protein family member HSPA2 was upregulated in rNS1-wt-infected cells at 24 hpi, while HSPA8 was upregulated in cells infected with either rNS1-wt or rNS1-SD30 viruses at 24 and 36 hpi. TRAP1, which is mitochondrial heat shock protein 75 (HSP75), is an anti-apoptotic/antioxidant protein that protects cells against apoptosis induced by tyrosine kinase inhibitors [50] and cisplatin [51]. Our results showed upregulation of TRAP1 in rNS1-wt and rNS1-SD30-infected cells at 24 hpi. The chaperonin containing TCP-1 complex (CCT; also known as TCP-1 ring complex, TRiC) has been implicated in the life cycle of several viruses. CCT specifically interacts with the PB2 subunit of the RNA polymerase, suggesting that it acts as a chaperone for PB2 to facilitate its folding and possibly its incorporation into the trimeric RNA polymerase complex [52]. In rNS1-wt-infected cells, CCT6A was downregulated at 12 hpi, while CCT7 was upregulated at 24 hpi. Since most HSPs positively regulate influenza virus replication, the fact that several HSPs were downregulated or unchanged in rNS1-SD30-infected cells seems to correlate with its weakened replication capacity and pathogenicity.

In conclusion, we provide the first quantitative proteomic study to systematically analyze the host response to recombinant H5N1 avian influenza viruses with intact and absent NS1 eIF4GI-binding domains. Using relevant *in vitro* models represented by primary and immortalized chicken fibroblasts, we identified numerous DE proteins likely associated with the distinct pathogenicity exhibited by each viral form. Overall, our data showed that the rNS1-wt virus induced larger changes at the proteomic level in host CEF cells compared with the NS1 mutant virus rNS1-SD30. We also identified several new proteins that may contribute to viral propagation. In particular, ANXA7 was shown to exert different effects on the propagation of each virus and on the IFN responses elicited by them. This finding suggests that ANXA7 is a potentially critical factor in the modulation of the immune response to H5N1 infection. Further studies are needed to elucidate in greater detail the functions of the proteins regulated by interaction of the viral eIF4GI-binding domain with the translational machinery of host cells.

## MATERIALS AND METHODS

### Cell culture and virus infection

Primary CEFs isolated from 10-day chicken embryos, the CEF cell line DF-1, and Madin-Darby Canine Kidney (MDCK) cells were grown in Dulbecco’s modified Eagle’s medium (DMEM; Gibco, Invitrogen) supplemented with 100 U/mL penicillin, 100 µg/mL streptomycin, and 10% FBS (Gibco, Invitrogen) at 37°C in an 5% CO_2_ incubator.

The recombinant H5N1 viruses rNS1-wt (HA, NA, and NS genes from H5N1 virus A/chicken/Hubei/327/2004, other genes from the A/WSN/33 virus) and rNS1-SD3(30 amino acids deleted in NS1 between residues 84 and 113) [11], were cultivated in 11-day chicken embryos and preserved at -80°C. For infection, cells were washed with PBS three times before culture in serum-free medium, and infected with these viruses at 0.01 or 1 MOI in DMEM containing 0.1 μg/mL of tosylsulfonyl phenylalanyl chloromethyl ketone (TPCK)-treated trypsin. All experiments with live H5N1 viruses were performed in BSL-3 facilities at Huazhong Agricultural University.

### Viral titer determination using plaque assays (PFU)

Cells’ supernatants containing rNS1-wt and rNS1-SD30 viruses were collected 12, 24, and 36 hpi. For plaque assays, confluent MDCK cells in 6- or 12-well plates were inoculated with appropriate dilutions of the harvested viruses in DMEM at 37°C for 1 h. Unbound viruses were removed by washing with PBS. Cells were then overlaid with 1.6% low-melting-temperature agarose (Amresco) mixed in a 1:1 ratio with 2× DEME without phenolsulfonphthalein (Hyclone) containing 3.7 g/L sodium bicarbonate (Amresco) and incubated at 37°C. After three days, agar was removed, and 0.1% crystal violet was added to facilitate plaque counting. Each test was repeated three times.

### Indirect immunofluorescence assay (IFA)

For indirect immunofluorescence staining, CEFs were grown on coverslips in 24-well plates. After 12, 24, and 36 hpi, cells were washed twice with PBS, fixed with 4% paraformaldehyde, and permeabilized with ice-cold acetone for 15 min. Then, nonspecific binding was blocked with 1% bovine serum albumin (BSA) in PBS at 37°C for 30 min before incubation with a mouse monoclonal antibody (prepared in our laboratory) against H5N1-NP protein at 37°C for 1 h. After five washes with PBS, the cells were incubated with FITC-labeled goat anti-mouse IgG (02-18-06, KPL, USA) at 37°C for 1 h in the dark. Cells were then washed 5 times with PBST and stained with 4′,6-diamidino-2-phenylindole-dihydrochloride (DAPI) to detect nuclei. Fluorescence was examined microscopically using an Olympus DP70 digital camera.

### Protein extraction

Cells grown in T-flasks (75 cm^2^) were identically processed for virus infection. Mock-infection was performed without supplemental virus in the control group to simulate the viral infection procedure. Proteins were respectively extracted at 12, 24, and 36 hpi as previously described [53]. At each time point, the medium was removed, and the cells were washed twice with PBS and transferred into sterile tubes containing 500 μL of lysis buffer (7 M urea, 2 M thiourea, 4% CHAPS, 1% DTT, and 2% IPG buffer, pH 4–7), with a proteinase inhibitor cocktail (0.5 mM AEBSF, 0.3 µM aprotinin, 10 µM bestatin, 10 µM E-64, and 10 µM leupeptin (Amresco)). The mixture was sonicated five times (10 s each time), incubated on ice for 60 min with vortexing every 15 min, and finally centrifuged at 12,000 ×*g* for 30 min at 4°C. The supernatant was collected, and protein concentration was determined using the PlusOne 2-D Quant Kit (GE Healthcare, USA).

### Two-dimensional electrophoresis (2-DE) and image analysis

Isoelectric focusing (IEF) was performed using the IPGphor II system (GE Healthcare) and Immobiline 24-cm DryStrip IPG strips (pH 4-7) as previously described [54]. Prepared proteins (150 mg/strip) were mixed with rehydration buffer (7 M urea, 2 M thiourea, 2% w/v CHAPS, 1% w/v DTT, and 0.5% v/v IPG buffer, pH 4-7, with 0.002% w/v bromophenol blue) and focused for 70 kV•h. After IEF, the IPG strips were equilibrated with 10 mg/mL of DTT and 40 mg/mL of iodoacetamide for 15 min in equilibration buffer (2% w/v SDS, 30% v/v glycerol, 0.002% w/v bromophenol blue, 6 M urea, and 50 mM Tris-HCl; pH 8.8). 2-DE was then performed on a 10% SDS-PAGE using an Ettan DALTSix electrophoresis unit (GE Healthcare). Prior to image analysis, background normalization was performed to adjust for differences in protein spot intensity. A 2-DE image of the control group was used as reference against which gel images of the infected groups were matched and contrasted. Percentage volume (%vol) and average intensity ratios were used for spot identification. DE protein spots with a change greater than 1.5-fold and a *p* < 0.05 were considered as upregulated or downregulated and selected for MS identification.

### In-gel protein digestion

As previously described [55], the protein spots were manually excised from the silver-stained gels and transferred to V-bottom 96-well microplates loaded with 100 µL of 50% ACN/25 mM ammonium bicarbonate solution per well. After being destained for 1 h, gel plugs were dehydrated with 100 µL of 100% ACN for 20 min and then thoroughly dried in a SpeedVac concentrator (Thermo Savant, USA) for 30 min. The dried gel particles were rehydrated at 4°C for 45 min with 2 µL/well of trypsin (Promega, Madison, WI, USA) in 25 mM ammonium bicarbonate and then incubated at 37°C for 12 h. After trypsin digestion, the peptide mixtures were extracted with 8 µL of extraction solution (50% ACN/0.5% TFA) per well at 37°C for 1 h prior to MALDI-TOF MS analysis.

### MALDI-TOF MS/MS analysis and database search

The MALDI matrix was prepared by dissolving α-cyano-4-hydroxycinnamic acid (Bruker Daltonics, Bremen, Germany) in 50% ACN and 0.1% TFA to make a 5 mg/ml solution. The solution was sonicated for 15 min and centrifuged at 10,000 rpm for 5 min. Mass spectra were obtained on an Ultraflex III MALDI TOF/TOF mass spectrometer (ABI4700, USA), in positive mode in the mass range of 700-3200 Da with an accelerating voltage of 20 kV. The Nd:YAG laser was operated at a wavelength of 355 nm. Two µL of peptide mixture was mixed with equal amount of matrix (clear supernatant) and 0.5 μl of this mix was spotted on a MALDI ground steel target plate. Peptide ion masses were internally calibrated using trypsin autolytic peptides at m/z 702.366 and 3419.542. MALDI analysis was performed by a fuzzy logic feedback control system (Ultraflex αMALDI TOF/TOF system Bruker, Germany) equipped with delayed ion extraction, applying the same peak detection and annotation parameters for both preparations. Parent mass peaks with mass range of 700-3200 Da and minimum signal-to-noise ratio of 50 were picked out for tandem TOF/TOF analysis. Protein identification was performed by searching extracted peak lists generated with one defined set of filter parameters against the NCBI database using the Mascot (Matrix Science, London, UK) search engine (http://www.matrixscience.com). Parameters and conditions were set as: tryptic digestion, a maximum of 2 missed cleavages, carbamidomethylation of cysteine as the fixed modification, oxidation of methionine as the variable modification, peptide mass tolerance of 50 ppm, and fragment MS tolerance of 0.2 Da. MASCOT protein scores (based on combined MS and MS/MS spectra) greater than 74 were considered statistically significant (*p* < 0.05).

### Protein network and function analyses

Differentially expressed proteins identified in this study were uploaded into the IPA application and mapped to the corresponding gi numbers in the IPA Knowledge Base. Possible connections between mapped proteins were evaluated based on curated interactions in the IPA database. Graphical networks of these proteins were then algorithmically generated. Proteins or protein products are represented as nodes, linked by their biological relationships. All linkages are supported by a minimum of one reference from the IPA literature or by canonical information stored in the IPA Knowledge Base. Nodes are displayed using different color intensities and shapes that represent relative abundance and functional classes of the gene product, respectively.

### Western blot analysis

Samples of influenza-infected and uninfected CEFs or DF-1 cells collected at 12, 24, and 36 hpi were lysed for total protein extraction, and protein concentration was determined as described above. Equivalent amounts of cell lysates (40 µg) were subjected to 10% SDS-PAGE and then transferred to nitrocellulose membranes (GE Healthcare, UK). After blocking with 1% BSA for 1 h at room temperature, the membranes were incubated with mouse monoclonal antibodies to human HSP70 (BM0368, BOSTER, China), PKM2 (ab218300, Abcam, USA), ENO1 (sc-100812, Santa Cruz, USA), and GAPDH (AC002, ABclonal, USA), and rabbit polyclonal antibodies to ANXA7 (A3733, ABclonal, USA), vimentin (2707-1, Epitomics, USA), NP (GTX125989, GeneTex, USA), and HA-tag (CB100005M, California Bioscience, USA). The membranes were then separately incubated with horseradish peroxidase (HRP)-conjugated anti-mouse IgG (074-1806, KPL, USA) and HRP-conjugated anti-rabbit IgG (074-1506, KPL, USA) for 1 h. After washing, bands were visualized with ECL (Advansta, USA) using a DNR Bio-Imaging System.

### Plasmid construction and transfection

A total of 26 full-length chicken genes for differentially expressed proteins (Supplementary Table 2) were amplified by PCR from the cDNA of influenza-infected or uninfected CEFs and inserted into pcDNA3.1-V5-His and pCAGGS-HA plasmids. The full-length chMDA5 was amplified from pcDNA6-chMDA5 (kindly provided by Nicolas Ruggli, Institute of Virology and Immunoprophylaxis (IVI), Mittelhäusern, Switzerland), and cloned into the plasmid pCAGGS-HA. The chicken IFN-β promoter sequence was cloned as described previously [56], inserted into the luciferase reporter plasmid pGL3-Basic (Promega), and designated as pGL3-chIFN-β-luc. NS1-wt and NS1-SD30 were inserted into the plasmid p3xFlag-CMV. Primers were designed using Primer Premier 5.0 software based on chicken gene sequences in GenBank. All constructions were confirmed by sequencing and western blot analysis upon transfection into DF-1 cells by using Lipofectamine 2000 reagent (Invitrogen, USA) according to the manufacturer’s recommendations for transient expression. In brief, cells were grown to 80% to 90% confluence in 6-, 12-, or 24-well culture plates. Plasmid DNA (1 μg) and Lipofectamine 2000 (2 μL) were diluted separately in Opti-MEM medium (Life Technologies), mixed together, and incubated for 5-10 min at room temperature. The DNA-Lipofectamine 2000 complex was then added to the cells. The cells were grown at 37°C for 24 h before infection or further processing.

### qRT-PCR

IFN-β mRNA levels induced by rNS1-wt and rNS1-SD30 viruses in infected CEFs were measured by qRT-PCR using SYBR green-based detection with an ABI PRISM 7500 cycler (Applied Biosystems, Foster City, CA, USA). CEF monolayers inoculated with the two rH5N1 variants for 12, 24, and 36 h were washed with ice-cold PBS and then lysed with TRIzol reagent (Invitrogen, USA). Total cellular RNA was extracted using the RNeasy Mini Kit (Qiagen, GmbH, Hilden, Germany) according to the manufacturer’s protocol. RNA concentrations were measured with a spectrophotometer (OD260/OD280). After DNA inactivation, 2 μg of RNA was reversely transcribed in a 20 μL reaction mixture containing 2 μL avian myeloblastosis virus (AMV) buffer, 50 pM Oligo18T, 0.5 mM dNTPs, 10 U RNase inhibitor, and 20 U AMV reverse transcriptase (TAKARA, Japan). After initial denaturation at 95°C for 30 s, amplification was carried out through 40 cycles, each consisting of denaturation at 95°C for 15 s, primer annealing at 60°C for 30 s, and DNA extension at 72°C for 45 s. Melting curves were obtained, and quantitative analysis of the data was performed using the 7500 System SDS software Version 1.3.1 using a relative quantification (ddCt) study model (Applied Biosystems). Primers for chicken IFN-β mRNA and the internal standard 18S rRNA were used as previously reported [16].

### Dual-luciferase reporter assay

DF-1 cells were seeded in 24-well plates and transfected the following day via standard Lipofectamine 2000 reagent transfection method. A firefly luciferase reporter plasmid pGL3-chIFN-β (100 ng/well) and a *Renilla* luciferase reporter plasmid pRL-TK (10 ng/well) were co-transfected with the indicated expression plasmids. In the same experiment, an empty control plasmid was added to ensure that the same amount of total DNA was transfected. At 24 h after transfection or further processing, dual-luciferase assays were performed using a dual-specific luciferase assay kit (Promega, Madison, WI, USA) according to the manufacturer’s protocol. Firefly luciferase activities were normalized based on *Renilla* luciferase activities. All reporter assays were repeated at least three times.

### Statistical analysis

Statistical analysis was performed using GraphPad Prism 5.0 (GraphPad Software, San Diego, CA, USA). Unless indicated otherwise, all data are presented as mean ± SD and analyzed using a two-tailed paired Student’s *t* test. Statistically significant differences between experimental groups were determined using two-way ANOVA. Significance was set as *p* < 0.05. All assays were performed in triplicate.

## SUPPLEMENTARY MATERIALS FIGURES AND TABLES











## Abbreviations

NS1: non-structural 1 protein
rNS1-SD30: Recombinant H5N1 virus lacking the eIF4GI-binding domain in NS1
rNS1-wt: Recombinant H5N1 virus with complete eIF4GI-binding domain in NS1
CEF: chicken embryo fibroblast
Hpi: hours post-infection
chMDA5: chicken Melanoma differentiation-associated protein 5
IAVs: Influenza A viruses
eIF4GI: eukaryotic translation initiation factor 4GI
RLR: retinoic acid-inducible gene I-like receptor
DE: differentially expressed
2-DE: 2-dimensional electrophoresis
IPA: Ingenuity Pathways Analysis
MOI: multiplicity of infection
CPE: cytopathic effect
qRT-PCR: quantitative real-time polymerase chain reaction
