# Supplementary material for: Proteomic analysis of chicken embryo fibroblast cells infected with recombinant H5N1 avian influenza viruses with and without NS1 eIF4GI binding domain
Source: Oncotarget. 2017 Dec 22;9(9):8350–67. doi: 10.18632/oncotarget.23615 (PMC5823584; doi:10.18632/oncotarget.23615)
Supplement: Supplementary file 1 [file oncotarget-09-8350-s001.pdf]

Proteomic analysis of chicken embryo fibroblast cells infected with recombinant H5N1 avian influenza viruses with and without NS1 eIF4GI binding domain

SUPPLEMENTARY MATERIALS

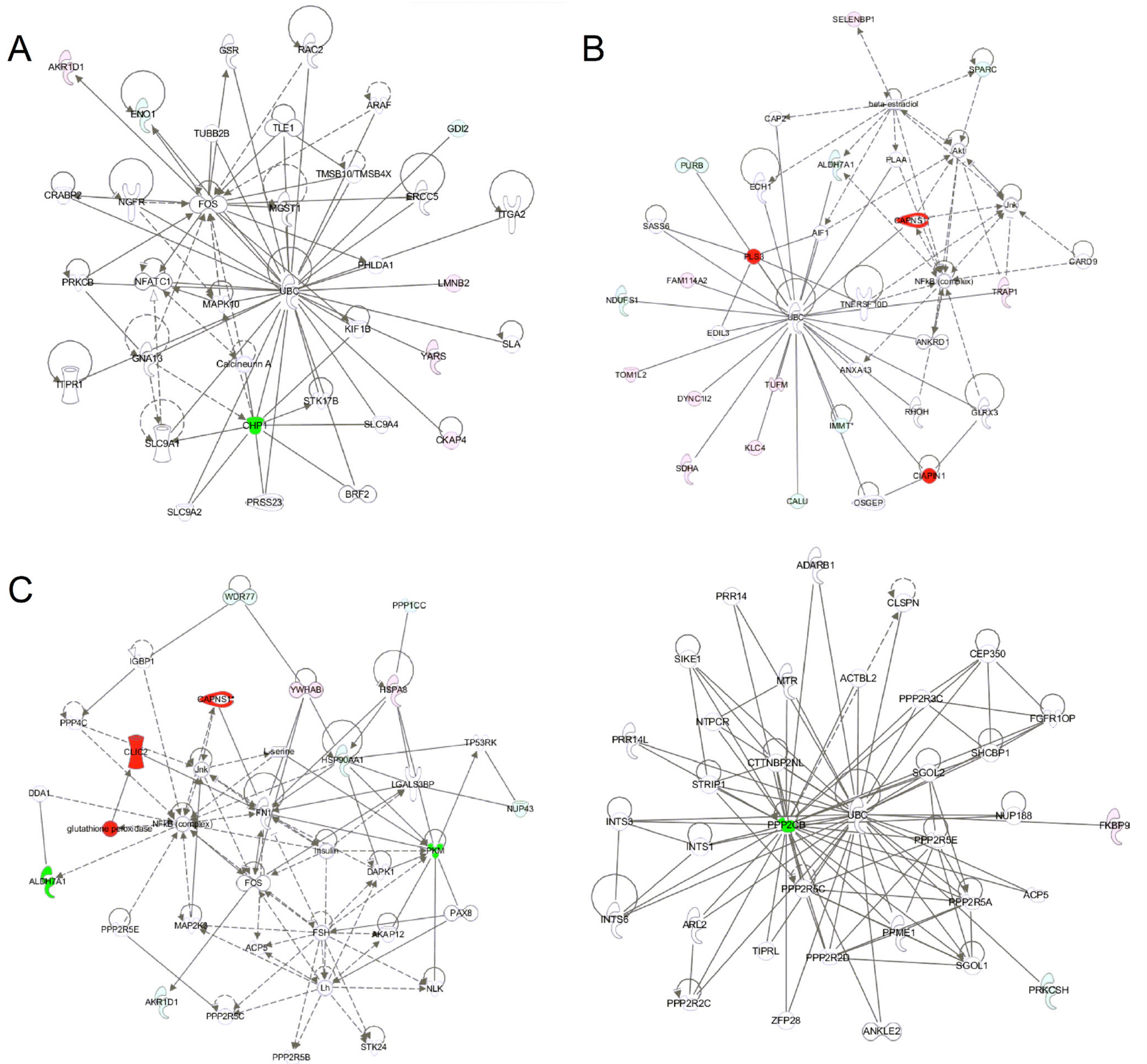

Supplementary Figure 1: Network pathway analysis protein identified by rNS1-wt relative to rNS1-SD30 infection. The other score network at 12 h (**A**), 24 h (**B**) and 36 h (**C**).

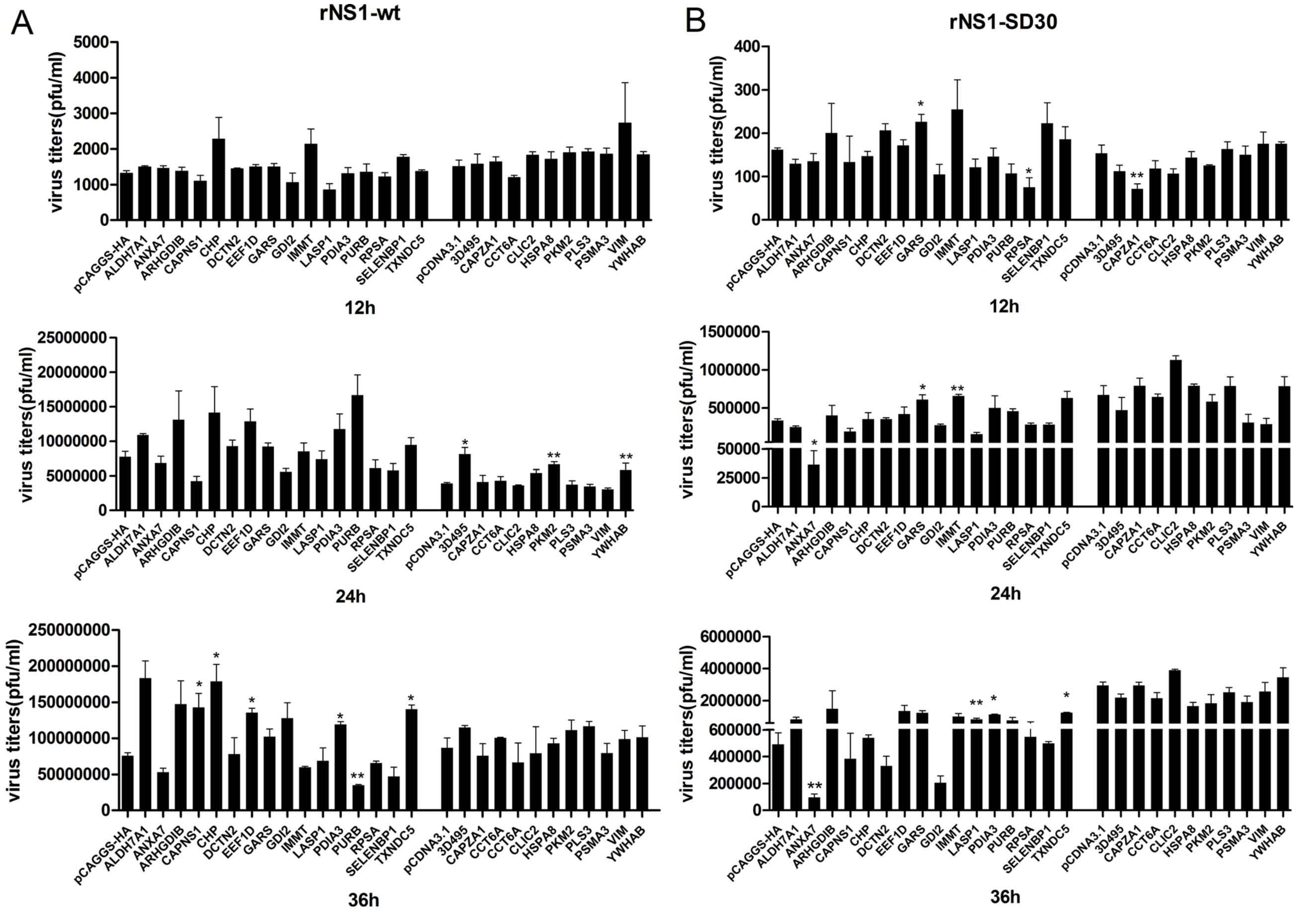

Supplementary Figure 2: The effect of overexpression DE proteins on rNS1-wt and rNS1-SD30 viruses replication. DF1 cells seeded in 12-well plate were transfected with 1µg control plasmid pCAGGS-HA or pCDNA3.1-V5 or indicated plasmids for 24 h, and infected with rNS1-wt (**A**) and rNS1-SD30 (**B**) virus at 0.01 MOI. Supernatant were collected at12, 24, and 36 hpi, and assessed for viral titers using plaque assays on MDCK cells.

Supplementary Table 1: The detailed functions of up-regulation and down-regulation proteins infected with each virus at each time point. See_Supplementary_Table 1

Supplementary Table 2: Primer sequences used for plasmids construction and qRT-PCR. See_ Supplementary_Table 2

Supplementary Table 3: Pairwise comparison of differentially expressed proteins in CEF at 24 hours post-inoculation with the H5N1 viruses rNS1-wt or rNS1-SD30. See_Supplementary_Table 3

Supplementary Table 4: 3 Pairwise comparison of differentially expressed proteins in CEF at 36 hours post-inoculation with the H5N1 virusesrNS1-wt or rNS1-SD30. See_Supplementary_Table 4
