# Supplementary material for: Proteomic analysis of chicken embryo fibroblast cells infected with recombinant H5N1 avian influenza viruses with and without NS1 eIF4GI binding domain
Source: Oncotarget. 2017 Dec 22;9(9):8350–67. doi: 10.18632/oncotarget.23615 (PMC5823584; doi:10.18632/oncotarget.23615)
Supplement: Supplementary file 2 [file oncotarget-09-8350-s002.doc]

**Supplementary Table 1:** The detailed functions of up-regulation and down-regulation proteins infected with each virus at each time point.

| **12 hpi** | | | | | |
| --- | --- | --- | --- | --- | --- |
| **rNS1-wt vs mock** | | **rNS1-SD30 vs mock** | | **rNS1-wt vs rNS1-SD30** | |
| **DE proteins** | **Function** | **DE proteins** | **Function** | **DE proteins** | **Function** |
| PSPC1 | transcription regulation | PSPC1 | transcription regulation | GDI2 | protein transport, signal transduction |
| CCT6A | molecular chaperone | DCTN2 | cell proliferation, mitosis | A2M | Protease inhibitor |
| GDI2 | protein transport, signal transduction | KRT19 | virus-host interaction | DCTN2 | cell proliferation, mitosis |
| A2M | protease inhibitor | A2M | signal transduction, protease inhibitor | ACTG1 | cytoskeleton |
| TOM1L2 | protein transport | LMNB2 | intermediate filament | YARS | protein biosynthesis |
| ANXA7 | social behavior, calcium-dependent phospholipid binding | RUVBL2 | Transcription regulation | CKAP4 | cytoskeleton |
| DCTN2 | cell proliferation, mitosis | ACTG1 | cytoskeleton | TXNDC5 | anti-apoptosis,cell redox homeostasis |
| KRT19 | virus-host interaction, response to estrogen stimulus | CHP1 | protein kinase inhibitor, protein transport | AKR1D1 | Lipid metabolism |
|  |  | LMNA | intermediate filament, apoptotic process | ENO1 | negative regulation of cell growth, response to virus |
|  |  |  |  | LASP1 | Ion transport |
|  | | | | | |
| **24 hpi** | | | | | |
| **rNS1-wt vs mock** | | **rNS1-SD30 vs mock** | | **rNS1-wt vs rNS1-SD30** | |
| **DE proteins** | **Function** | **DE proteins** | **Function** | **DE proteins** | **Function** |
| ACTG1 | cytoskeleton | ACTG1 | cytoskeleton | SPARC | regulation of cell proliferation |
| PKM2 | programmed cell death, small molecule metabolic process | PKM2 | programmed cell death, metabolic process | LMNA | intermediate filament, apoptotic process |
| CKAP4 | cytoskeleton | RPSA | cell adhesion, catabolic process, ribosomal assembly, translation | CAPNS1 | cytoskeletal remodeling, signal transduction |
| PURB | transcription regulation | SPARC | regulation of cell proliferation | ALDH7A1 | cellular aldehyde metabolic process |
| RPSA | cell adhesion, mRNA catabolic process, translation | IMMT | mitochondrial calcium ion homeostasis | GDI2 | protein transport, signal transduction |
| SPARC | regulation of cell proliferation | TRAP1 | cellular response to oxidative stress, chaperone | DCTN2 | cell proliferation, mitosis |
| TPM3 | cytoskeleton | KLC4 | cytoskeleton, organelle transport | CALU | platelet activation |
| IMMT | mitochondrial calcium ion homeostasis | VIM | cytoskeleton，Intermediate filament proteins | PDIA3 | cell redox homeostasis, signal transduction |
| LMNA | Intermediate filament, apoptotic process | PPP1CC | metabolic process, cell division | EEF1D | positive regulation of I-κB kinase/NF-κB |
| HSPA2 | Stress response | NDUFS1 | electron transport, apoptotic process |  |  |
| SDHA | electron transport, succinate metabolic process | TOM1L2 | protein transport |  |  |
| TRAP1 | cellular response to oxidative stress, chaperone | CIAPIN1 | negative regulation of apoptotic process |  |  |
| PLS3 | actin-binding and calcium ion binding | ALDH7A1 | cellular aldehyde metabolic process |  |  |
| DPYSL2 | cytoskeleton organization, differentiation | PSMA3 | interaction between organisms, apoptotic process |  |  |
| FAM114A2 | purine nucleotide binding | LMNB2 | intermediate filament |  |  |
| KLC4 | cytoskeleton, organelle transport | GDI2 | protein transport, signal transduction |  |  |
| TCP1/CCT7 | molecular chaperone | GARS | protein biosynthesis, cell death |  |  |
| SELENBP1 | protein transport | HSPA8 | regulation of cell cycle, molecular chaperone |  |  |
| TXNDC5 | anti-apoptosis, cell redox homeostasis |  |  |  |  |
| TUFM | protein biosynthesis, translation elongation factor activity |  |  |  |  |
| VIM | cytoskeleton, Intermediate filament proteins |  |  |  |  |
| SEPT2 | cell cycle and division, mitosis |  |  |  |  |
| CNN3 | negative regulation of ATPase activity |  |  |  |  |
| PPP1CC | carbohydrate metabolic process, cell division |  |  |  |  |
| TPM4 | stress response，Cytoskeleton |  |  |  |  |
| NDUFS1 | respiratory chain electron transport, apoptotic process |  |  |  |  |
| DYNC1I2 | host-virus interaction, transport |  |  |  |  |
| ACTR1A | cytoskeleton |  |  |  |  |
| GDI2 | protein transport, signal transduction |  |  |  |  |
| CAPNS1 | cytoskeletal remodeling, signal transduction |  |  |  |  |
| HSPA8 | regulation of cell cycle, molecular chaperone |  |  |  |  |
|  | | | | | |
| **36 hpi** | | | | | |
| **rNS1-wt vs mock** | | **rNS1-SD30 vs mock** | | **rNS1-wt vs rNS1-SD30** | |
| **DE proteins** | **Function** | **DE proteins** | **Function** | **DE proteins** | **Function** |
| TGM2 | apoptotic cell clearance and induction of apoptosis | PDIA4 | cell redox homeostasis | A2M | signal transduction, protease inhibitor |
| PRKCSH | N-glycan processing | VIM | cytoskeleton, Intermediate filament proteins | CHP1 | negative regulation NF-κB transcription, exocytosis |
| PDIA4 | cell redox homeostasis | FKBP9 | accelerate the folding of proteins during protein synthesis | PPP2CB | hydrolase, protein phosphatase |
| VIM | cytoskeleton, Intermediate filament proteins | PRKAR1A | signal transduction | KRT8 | cytoskeleton organization, virus-host interaction |
| FKBP9 | accelerate the folding of proteins during protein synthesis | RNH1 | mRNA catabolic process, regulation of angiogenesis | DCTN2 | cell proliferation, mitosis |
| PKM2 | programmed cell death, small molecule metabolic process | ATXN3 | Stress response | TPM3 | cytoskeleton |
| PRKAR1A | signal transduction | RPSA | cell adhesion, catabolic process, ribosomal assembly, translation | TGM2 | apoptotic cell clearance and induction of apoptosis |
| RNH1 | mRNA catabolic process, regulation of angiogenesis | SPARC | regulation of cell proliferation | PRKCSH | N-glycan processing |
| Nup43 | cell division, mRNA transport, cell cycle | FNTA | prenyltransferase, Transferase | WDR61 | wnt receptor signalling pathway, Transcription regulation |
| WDR77 | transcription regulation | STRAP | mRNA processing and splicing | CAPNS1 | cytoskeletal remodeling , signal transduction |
| ATXN3 | stress response | HNRPC | mRNA processing and splicing | GAPDH | apoptosis, Glycolysis |
| RPSA | cell adhesion, catabolic process, ribosomal assembly , translation | KPNB1 | protein transport, virus-host interaction | CKAP4 | cytoskeleton |
| SPARC | regulation of cell proliferation | TPM1 | cytoskeleton | KRT19 | virus-host interaction, response to estrogen stimulus |
| FNTA | prenyltransferase, Transferase | TPM4 | stress response，cytoskeleton | ALDH7A1 | cellular aldehyde metabolic process |
| SAE1 | protein sumoylation, protein ubiquitination | ACTG1 | cytoskeleton | 3D495 | methyltransferase activity |
| CNN3 | negative regulation of ATPase activity | PPP1CC | carbohydrate metabolism, cell division, glycogen metabolic process | GDI2 | protein transport, signal transduction |
| STRAP | mRNA processing and splicing | CAPNS1 | cytoskeletal remodeling, signal transduction |  |  |
| HNRPC | mRNA processing and splicing | CKAP4 | cytoskeleton |  |  |
| WDR61 | wnt receptor signaling pathway, Transcription regulation | KRT19 | virus-host interaction, response to estrogen stimulus |  |  |
| KPNB1 | Protein transport, virus-host interaction | LMNA | Intermediate filament, apoptotic process |  |  |
| TPM1 | cytoskeleton | ALDH7A1 | cellular aldehyde metabolic process |  |  |
| TPM4 | stress response, cytoskeleton | CAPZA1 | cytoskeleton organization |  |  |
| NACA | protein transport, transcription, DNA-dependent | 3D495 | methyltransferase activity |  |  |
| AKR1D1 | lipid metabolism | PDHB | acetyl-CoA biosynthetic process |  |  |
| CSTF2 | mRNA processing | GDI2 | protein transport, signal transduction |  |  |
| ACTG1 | cytoskeleton | HSP90AA1 | chaperone, stress response |  |  |
| PPP1CC | carbohydrate metabolism, cell division, glycogen metabolic process |  |  |  |  |
| YWHAB | activation of pro-apoptotic signaling and MAPKK activity |  |  |  |  |
| LMNB2 | Intermediate filament |  |  |  |  |
| CAPNS1 | cytoskeletal remodeling, signal transduction |  |  |  |  |
| CLIC2 | Ion transport |  |  |  |  |
| GAPDH | apoptosis, glycolysis |  |  |  |  |
| HSP90AA1 | chaperone, stress response |  |  |  |  |

Green, down-regulated proteins; red, up-regulated proteins.
