# Supplementary material for: Proteomic analysis of chicken embryo fibroblast cells infected with recombinant H5N1 avian influenza viruses with and without NS1 eIF4GI binding domain
Source: Oncotarget. 2017 Dec 22;9(9):8350–67. doi: 10.18632/oncotarget.23615 (PMC5823584; doi:10.18632/oncotarget.23615)
Supplement: Supplementary file 3 [file oncotarget-09-8350-s003.doc]

**Supplementary Table 2:** Primer sequences used for plasmids construction and qRT-PCR

| **Primer sequences used for plasmid constructions** | | | |
| --- | --- | --- | --- |
| **pCAGGS-HA** | | | |
| Gene Name | GenBank Accession No. | Primer Sequence (5’–3’) | |
| ALDH7A1-F | XM_424422.2 | | ccATCGATatgctcggttggcgccg |
| ALDH7A1-R |  |  | ccCTCGAGctgaaacttgattccttgagccaaag |
| ANAX7-F | NM_001277347.1 | | ccATCGATatggcatacccaggttacccc |
| ANAX7-R |  |  | ccCTCGAGttgaccaacaattgccagtagca |
| ARHGDIB-F | NM_001277364.1 | | cGAGCTCatgactgagaagacccaggaacc |
| ARHGDIB-R |  |  | ggGGTACCttctgtccattccttcttgatgg |
| ANAX7-F | NM_001277347.1 | | ccATCGATatggcatacccaggttacccc |
| ANAX7-R |  |  | ccCTCGAGttgaccaacaattgccagtagca |
| CAPNS1-F | XM_001232968.2 | | ccATCGATatgtttcttgctaaggctttg |
| CAPNS1-R |  |  | ccCTCGAGtcacgaatacatggtcagct |
| CHP1-F | NM_001007930.1 | | ccATCGATatgggttcccgagcgtctac |
| CHP1-R |  |  | ccCTCGAGgtgaagaaatcgaatgctcatt ttc |
| DCTN2-F | NM_204802.1 | | ccATCGATatggccgaccccaaatacg |
| DCTN2-R |  |  | ccCTCGAGctgcagccgcttgatgcg |
| EEF1D-F | XM_001232627.1 | | ccATCGATatgaggaccaggaagcctcc |
| EEF1D-R |  |  | ccCTCGAGgatcttgttgaaagcagcaatgtc |
| GARS-F | NM_001031510.1 | | ccATCGATatggacggcccgcaagc |
| GARS-R |  |  | ccCTCGAGttcctctattgtgtcctttttgcca |
| GDI2-F | NM_205004.1 | | ccGAATTCatgaatgaggagtacgacgtg |
| GDI2-R |  |  | ccCTCGAGctgctgctcctcctccc |
| IMMT-F | NM_001006462.1 | | ccATCGATatgctgcgggcctgtcag |
| IMMT-R |  |  | gaAGATCTctgcacttgagttgttcccaacc |
| LASP1-F | NM_001177329.1 | | ccATCGATatgaaccccaactgcgcg |
| LASP1-R |  |  | ccCTCGAGgatggcctcaacgtagttggc |
| PDIA3-F | NM_204110.3 | | ccATCGATatgtccgtgcctcggcc |
| PDIA3-R |  |  | ccCTCGAGcaaatcctcctttgccttcttct |
| PURB-F | AY706044.1 | | ccATCGATatggcgaccccctg |
| PURB-R |  |  | ccCTCGAGttaaagtgtcagctcaat |
| RPSA-F | NM_001007823.1 | | ccATCGATatgtccggaggtctcgatgtcc |
| RPSA-R |  |  | ccCTCGAGagaccactccgtggtagtcccaa |
| SELENBP1-F | XM_423397.2 | | ccATCGATatggcaaaatgcggagcg |
| SELENBP1-R |  |  | ccCTCGAGtatccagatgtccgaggtgctg |
| TXNDC5-F | NM_001277810.1 | | ccATCGATatggcttcgctgcgctcc |
| TXNDC5-R |  |  | ccCTCGAGcaattcatcccttgcctgacg |
| MDA5-F | GU570144.1 | | ccATCGATatgtcggaggagtgccgag |
| MDA5-R |  |  | cgGCTAGCatcttcatcacttgaaggacaatgag |
| **pcDNA3.1-V5-His** | | | |
| 3D495-F | XM_001232693.3 | | aaaGGATCCatggccacgcagatgtttgag |
| 3D495-R |  |  | aaaCTCGAGcgcttccttgtgccccagg |
| CAPZA1-F | NM_205515.1 | | aaaGGATCCatggccgactttgaggaccg |
| CAPZA1-R |  |  | aaaCTCGAGagcattctgcatttctttgccaat |
| CLIC2-F | NM_001031114.1 | | aaaGGATCCatggagagccggccggtg |
| CLIC2-R |  |  | aaaCTCGAGggtcatcttcctggccacgct |
| HSPA8-F | NM_205003.2 | | aaaAAGCTTatgtcaaagggaccagctgttggtatc |
| HSPA8-R |  |  | aaaTCTAGAatccacctcctcaatggttggtcc |
| PKM2-F | NM_205469.1 | | ccATCGATatgtcgaagcaccacgatgc |
| PKM2-R |  |  | ccCTCGAGtcatggcacgggcacca |
| PLS3-F | NM_001006431.1 | | aaaGGATCCatggctgtcaccatgcagatctcc |
| PLS3-R |  |  | aaaCTCGAGtactcgcttcatccctctgccca |
| PSMA3-F | NM_001006491.1 | | ataGGATCCatgagctccatcggcaccgg |
| PSMA3-R |  |  | ataCTCGAGcatattttcatcatctgattcatcttcctcttt |
| VIM-F | NM_001048076.1 | | aaaGGATCCatgagcttcaccagcagcaagaact |
| VIM-R |  |  | aaaCTCGAGctccaagtcatcgtgatgctggg |
| YWHAE-F | NM_001006219.1 | | cgGGATCCatggacgatcgggaggacct |
| YWHAE-R |  |  | tttCGAGTGctggttttcatcttccacatcctg |
| **pGL3-Basic** | | | |
| IFN-β-F | Y14969.1 | | ggCTCGAGcagccaccacatggtctcacct |
| IFN-β-R |  |  | tttAAGCTTaaccttggtgggacttgtgttctg |
| **p3xFlag-CMV** | | | |
| NS1-F | AY684710.1 | | GGAATTCACCatggattccaacactgtgt |
| NS1-R |  |  | CGGGATCCaacttttgactcaattgttctc |
| **Primer sequences used for qRT-PCR** | | | |
| IFN-β-F | GU119897.1 | | CCTCCAACACCTCTTCAACATG |
| IFN-β-R |  |  | TGGCGTGCGGTCAAT |
| 18S-F | AF173612.1 | | TCCCCTCCCGTTACTTGGAT |
| 18S-R |  |  | GCGCTCGTCGGCATGTA |
