# Supplementary material for: Proteomic analysis of chicken embryo fibroblast cells infected with recombinant H5N1 avian influenza viruses with and without NS1 eIF4GI binding domain
Source: Oncotarget. 2017 Dec 22;9(9):8350–67. doi: 10.18632/oncotarget.23615 (PMC5823584; doi:10.18632/oncotarget.23615)
Supplement: Supplementary file 4 [file oncotarget-09-8350-s004.docx]

**Supplementary Table 3: Pairwise comparison of differentially expressed proteins in CEF at 24 hours post-inoculation with the H5N1 viruses rNS1-wt or rNS1-SD30.**

| **Spot**  **ID^a^** | **Protein name**  **(Abbreviation)** | **Accession**  **No^b^** | **Differentially expressed proteins identified in CEF between groups** | | | | | | **Protein**  **score^d^** | **Matched peptide^e^** |
| --- | --- | --- | --- | --- | --- | --- | --- | --- | --- | --- |
|  |  |  | **rNS1-wt and Mock** | | **rNS1-SD30 and Mock** | | **rNS1-wt and rNS1-SD30** | |  |  |
|  |  |  | ***P-*value** | **Ratio^c^** | ***P-*value** | **Ratio** | ***P-*value** | **Ratio** |  |  |
| 17-01 | actin, cytoplasmic type5(ACTG1) | gi\|56119084 | 4.5e-17 | -100^f^ | - | -^g^ | - | - | 225 | 6 |
| 17-03 | Pyruvate kinase PKM (PKM2) | gi\|45382651 | 4.8e-14 | -100 | 4.8e-14 | -100 | - | - | 86 | 9 |
| 17-04 | cytoskeleton-associated protein 4(CKAP4) | gi\|118082813 | 5.8e-15 | -2.07 | - | - | - | - | 204 | 8 |
| 17-05 | transcriptional activator protein Pur-beta(PURB) | gi\|74228215 | 0.00021 | -7.14 | - | - | - | - | 98 | 2 |
| 17-06 | 40S ribosomal protein SA(RPSA) | gi\|308081909 | 9.1e-36 | -6.37 | 9.1e-36 | -100 | - | - | 412 | 8 |
| 17-07 | SPARCprecursor(SPARC) | gi\|45383337 | 5.8e-16 | -3.19 | 5.8e-16 | -1.72 | 5.8e-16 | -1.85 | 214 | 7 |
| 17-08 | tropomyosin 3（TPM3） | gi\|53129586 | 9.1e-15 | -100 | - | - | - | - | 202 | 11 |
| 18-05 | Mitochondrial inner membrane protein (IMMT) | gi\|57530041 | 2.9e-22 | 2.34 | - | - | - | - | 277 | 12 |
| 18-11 | lamin-A (LMNA) | gi\|45384214 | 2.9e-25 | 1.7- | - | - | 2.9e-25 | 1.13- | 307 | 22 |
| 18-12 | heat shock 70 kDa protein2(HSPA2) | gi\|55742654 | 0.00024 | 2.2- | - | - | - | - | 98 | 6 |
| 18-13 | Succinate dehydrogenase Fp subunit(SDHA) | gi\|3851616 | 9.1e-07 | 1.53 | - | - | - | - | 122 | 7 |
| 18-14 | TNF receptor-associated protein 1(TRAP1) | gi\|57525126 | 4.6e-17 | 3.15 | 4.6e-17 | 2.75 | - | - | 225 | 10 |
| 18-15 | ChainA,Crystal Structure Of Hsc70BAG1 IN COMPLEX WITH ATP9(HSPA8) | gi\|225698069 | 4.5e-11 | 2.57 | 4.5e-11 | 2.54 | - | - | 165 | 8 |
| 18-19 | plastin-3(PLS3) | gi\|57530180 | 1.4e-26 | 100 | - | - | - | - | 320 | 11 |
| 18-22 | dihydropyrimidinase-related protein 2(DPYSL2) | gi\|45383177 | 0.0012 | 2.02 | - | - | - | - | 91 | 7 |
| 18-24 | Protein FAM114A2  (FAM114A2) | gi\|50755009 | 1.1e-21 | 1.7 | - | - | - | - | 271 | 5 |
| 18-25 | kinesin light chain4-like(KLC4) | gi\|118087579 | 0.0018 | 5.94 | 0.0018 | 3.77 | - | - | 89 | 5 |
| 18-26 | T-complex protein 1 subunit eta (CCT7) | gi\|71895883 | 7.2e-29 | 1.58 | - | - | - | - | 343 | 14 |
| 18-27 | Pyruvate kinase PKM (PKM2) | gi\|45382651 | 5.7e-07 | 1.69 | - | - | 5.7e-07 | 2.23 | 124 | 7 |
| 18-32 | selenium-binding protein 1 (SELENBP1) | gi\|118102241 | 1.1e-06 | 2.15 | - | - | - | - | 121 | 4 |
| 18-35 | thioredoxin domain-containing protein 5(TXNDC5) | gi\|57530789 | 1.1e-22 | 1.80 | - | - | - | - | 281 | 6 |
| 18-36 | Elongation factor Tu, mitochondrial(TUFM) | gi\|88909611 | 2.9e-25 | 1.71 |  | - | - | - | 307 | 10 |
| 18-37 | Vimentin(VIM) | gi\|114326309 | 5.8e-32 | 2.93 | 5.8e-32 | 2.89 | - | - | 374 | 15 |
| 18-38 | **Septin-2(**SEPT2) | gi\|50752104 | 1.8e-10 | 1.80 | - | - | - | - | 159 | 5 |
| 18-40 | actin, cytoplasmic type 5(ACTG1) | gi\|56119084 | 9.1e-18 | 100 | - | - | - | - | 232 | 4 |
| 18-41 | Calponin 3, acidic（CNN3） | gi\|50751284 | 4.6e-09 | 1.69 | - | - | - | - | 145 | 10 |
| 18-43 | serine/threonine-protein phosphatase PP1-gamma catalytic subunit(PPP1CC) | gi\|57525187 | 2.9e-19 | 4.38 | 2.9e-19 | 5.10 | - | - | 247 | 12 |
| 18-44 | Tropomyosin-4(TPM4) | gi\|515694 | 7.2e-39 | 100 | - | - | - | - | 443 | 14 |
| 18-49 | NADH-ubiquinone oxidoreductase 75 kDa subunit (NDUFS1) | gi\|57529753 | 1.1e-15 | 1.47 | 1.1e-15 | 1.63 | - | - | 211 | 14 |
| 18-52 | Cytoplasmic dynein 1 intermediate chain 2（**DYNC1I2**） | gi\|55726736 | 3.6e-14 | 1.50 | - | - | - | - | 196 | 6 |
| 18-63 | alpha-centractin  (ACTR1A) | gi\|56118984 | 1.4e-12 | 100 | - | - | - | - | 180 | 5 |
| 18-65 | rab GDP dissociation inhibitor beta(GDI2) | gi\|45384364 | 2.3e-05 | 1.37 | - | - | - | - | 108 | 7 |
| 18-66 | vimentin(VIM) | gi\|57240090 | 3.6e-37 | 4.08 | 3.6e-37 | 2.92 | - | - | 426 | 15 |
| 18-72 | calpain small subunit  (CAPNS1 ) | gi\|2506056 | 1.1e-05 | 5.12 | - | - | 1.1e-05 | 8.51 | 111 | 2 |
| 18-75 | vimentin(VIM) | gi\|57240089 | 3.6e-19 | 3.47 | - | - | - | - | 246 | 7 |
| 19-01 | TOM1-like protein 2 (TOM1L2) | gi\|118097857 | - | - | 2.9e-15 | -2.16 | - | - | 207 | 10 |
| 19-07 | Anamorsin(CIAPIN1) | gi\|57524844 | - | - | 4.6e-12 | -100 | - | - | 175 | 4 |
| 20-02 | mitochondrial inner membrane protein(IMMT) | gi\|57530041 | - | - | 1.1e-57 | 3.31 | - | - | 631 | 19 |
| 20-11 | dehydrogenase 7 family, member A1  (ALDH7A1) | gi\|118104602 | - | - | 0.015 | 7.36 | 0.015 | -3.98 | 80 | 5 |
| 20-17 | serine/threonine-protein phosphatase PP1-gamma catalytic subunit(PPP1CC) | gi\|345323276 | - | - | 1.8e-22 | 2.02 | - | - | 279 | 14 |
| 20-21 | proteasome subunit alpha type-3(PSMA3) | gi\|57529899 | - | - | 3.6e-21 | 2.19 | - | - | 266 | 11 |
| 20-23 | lamin-B2(LMNB2) | gi\|45384202 | - | - | 9.1e-16 | 4.67 | - | - | 212 | 11 |
| 20-25 | rho GDP-dissociation inhibitor 2 (ARHGDIB) | gi\|50728568 | - | - | 1.1e-12 | 100 | 1.1e-12 | -100 | 181 | 5 |
| 20-27 | glycyl-tRNA synthetase(GARS) | gi\|71895709 | - | - | 2.9e-22 | 3.99 | - | - | 277 | 20 |
| 20-32 | beta-actin FE-3(**ACTG1** ) | gi\|13516471 | - | - | 0.0024 | 1.86 | 0.0024 | 2.28 | 88 | 3 |
| 23-12 | calpain small subunit(CAPNS1) | gi\|2506056 | - | - | - | - | 3.6e-28 | 100 | 336 | 3 |
| 24-03 | dynactin subunit 2(DCTN2) | gi\|45382201 | - | -- | - | - | 1.8e-36 | -1.83 | 419 | 12 |
| 24-05 | calumenin precursor(CALU) | gi\|47498076 | - | - | - | - | 3.6e-20 | -1.57 | 256 | 5 |
| 24-09 | protein disulfide-isomerase A3 precursor(PDIA3) | gi\|45383890 | - | - | - | - | 7.2e-06 | -100 | 113 | 6 |
| 24-10 | Eukaryotic translation elongation factor 1 delta(EEF1D) | gi\|118087445 | - | - | - | - | 2.9e-13 | -100 | 187 | 6 |

**^a^** to **^g^** refer to the corresponding footnotes in table 1.
