# Supplementary material for: Proteomic analysis of chicken embryo fibroblast cells infected with recombinant H5N1 avian influenza viruses with and without NS1 eIF4GI binding domain
Source: Oncotarget. 2017 Dec 22;9(9):8350–67. doi: 10.18632/oncotarget.23615 (PMC5823584; doi:10.18632/oncotarget.23615)
Supplement: Supplementary file 5 [file oncotarget-09-8350-s005.docx]

**Supplementary Table 4: Pairwise comparison of differentially expressed proteins in CEF at 36 hours post-inoculation with the H5N1 virusesrNS1-wt or rNS1-SD30.**

| **Spot**  **ID^a^** | **Protein name**  **(Abbreviation)** | **Accession**  **No^b^** | **Differentially expressed proteins identified in CEF between groups** | | | | | | **Protein**  **score^d^** | **Matched peptide^e^** |
| --- | --- | --- | --- | --- | --- | --- | --- | --- | --- | --- |
|  |  |  | **rNS1-wt and Mock** | | **rNS1-SD30 and Mock** | | **rNS1-wt and rNS1-SD30** | |  |  |
|  |  |  | ***P-*value** | **Ratio^c^** | ***P-*value** | **Ratio** | ***P-*value** | **Ratio** |  |  |
| 25-01 | heat shock protein HSP 90-alpha (HSP90AA1) | gi\|157954047 | - | -^g^ | - | - | 7.2e-18 | 2.98 | 233 | 8 |
| 25-09 | calcium-binding protein p22(CHP) | gi\|56118996 | - | - | - | - | 3.6e-21 | 1.50 | 266 | 5 |
| 25-10 | serine/threonine-protein phosphatase 2A catalytic subunit alpha isoform(PPP2CB) | gi\|45384108 | - | - | - | - | 1.8e-14 | 100^f^ | 199 | 5 |
| 26-07 | Vimentin(VIM) | gi\|114326309 | - | - | - | - | 2.9e-13 | -1.61 | 187 | 15 |
| 26-10 | cytokeratin 8(KRT8) | gi\|118129654 | - | - | - | - | 0.00015 | -1.90 | 79 | 3 |
| 26-12 | dynactin subunit 2(DCTN2) | gi\|45382201 | - | - | - | - | 1.8e-36 | -100 | 419 | 12 |
| 26-14 | SUMO-activating enzyme subunit 1-like, partial  (SAE1) | gi\|118125845 | - | - | - | - | 0.00015 | -3.18 | 100^f^ | 2 |
| 26-17 | tropomyosin 3 （TPM3） | gi\|224084038 | - | - | - | - | 7.2e-18 | -1.69 | 233 | 13 |
| 26-20 | rho GDP-dissociation inhibitor 2(ARHGDIB) | gi\|50728568 | - | - | 1.1e-12 | 3.53 | 1.1e-12 | -11.92 | 181 | 5 |
| 33-03 | heat shock protein HSP 90-alpha（HSP90AA1） | gi\|157954047 | 7.2e-20 | -1.91 | 7.2e-20 | -1.28 | - | - | 253 | 8 |
| 33-05 | Protein-glutamine gamma-glutamyltransferase 2 (TGM2) | gi\|45382075 | 9.1e-15 | -1.99 | - | - | 9.1e-15 | -1.70 | 202 | 8 |
| 33-08 | Protein disulfide-  isomerase A4(PDIA4) | gi\|57530768 | 1.4e-13 | -2.37 | 1.4e-13 | -3.18 | - | - | 190 | 11 |
| 33-09 | glucosidase 2 subunit beta-like（PRKCSH） | gi\|345326432 | 0.0024 | -3.48 | - | - | 0.0024 | -1.90 | 86 | 4 |
| 33-13 | Vimentin(VIM) | gi\|114326309 | 7.2e-25 | -1.59 | 7.2e-25 | -1.64 | - | - | 303 | 8 |
| 33-14 | peptidyl-prolyl cis-trans isomerase (FKBP9) | [gi\|45382327](http://www.matrixscience.com/cgi/protein_view.pl?file=../data/20110916/FtocmzHne.dat&hit=1) | 5.8e-27 | -1.60 | 5.8e-27 | -1.62 | - | - | 324 | 9 |
| 33-17 | Pyruvate kinase PKM (PKM2) | gi\|45382651 | 4.8e-14 | -100 | - | - | - | - | 86 | 9 |
| 33-20 | cAMP-dependent protein kinase type I-alpha regulatory subunit(PRKAR1A) | gi\|56119042 | 7.2e-21 | -1.95 | 7.2e-21 | -1.61 | - | - | 263 | 5 |
| 33-23 | ribonuclease inhibitor(RNH1) | gi\|57529989 | 1.1e-15 | -1.64 | 1.1e-15 | -1.06 | - | - | 211 | 3 |
| 33-27 | Nucleoporin 43（NUP43） | gi\|118088359 | 1.4e-13 | -1.96 | - | - | - | - | 190 | 8 |
| 33-28 | Methylosome protein 50 (WDR77) | gi\|71895697 | 1.8e-34 | -1.96 | - | - | - | - | 399 | 8 |
| 33-29 | ataxin-3(ATXN3) | gi\|45383440 | 1.8e-09 | -1.64 | 1.8e-09 | -2.48 | - | - | 149 | 7 |
| 33-30 | 40S ribosomal protein SA(RPSA) | gi\|308081909 | 9.1e-36 | -3.07 | 9.1e-36 | -17.07 | - | - | 412 | 8 |
| 33-31 | SPARC precursor(SPARC) | gi\|45383337 | 5.8e-16 | -3.49 | 5.8e-16 | -3.15 | - | - | 214 | 7 |
| 33-32 | farnesyltransferase alpha  (FNTA) | gi\|118104309 | 2.3e-17 | -2.06 | 2.3e-17 | -1.65 | - | - | 228 | 6 |
| 33-33 | SUMO-activating enzyme subunit 1-like, partial  (SAE1) | gi\|118125845 | 0.00015 | -2.53 | - | - | - | - | 100 | 2 |
| 33-35 | Calponin 3, acidic（CNN3） | gi\|50751284 | 5.8e-07 | -1.50 | - | - | - | - | 124 | 11 |
| 33-36 | serine-threonine kinase receptor-associated protein（STRAP） | gi\|57525428 | 7.2e-10 | -2.06 | 7.2e-10 | -1.01 | - | - | 153 | 3 |
| 33-37 | heterogeneous nuclear ribonucleoprotein C (C1/C2), isoform CRA_c(HNRPC) | gi\|119586801 | 0.00017 | -4.35 | - | - | - | - | 99 | 3 |
| 33-40 | WD repeat-containing protein 61(WDR61) | gi\|94536819 | 0.00023 | -2.10 | - | - | 0.0002 | 1.69 | 98 | 5 |
| 33-41 | karyopherin (importin) beta 1, isoform CRA_b(KPNB1) | gi\|119615215 | 4.5e-29 | -2.10 | 4.5e-29 | -2.06 | - | - | 345 | 17 |
| 33-44 | alpha-tropomyosin 2  (TPM2 ) | gi\|27465053 | 5.8e-25 | -1.77 | 5.8e-2 | -1.33 | - | - | 304 | 12 |
| 33-46 | Tropomyosin（TPM4） | gi\|515694 | 7.2e-11 | -1.45 | 7.2e-11 | -1.70 | - | - | 163 | 4 |
| 33-47 | Nascent polypeptide-  associated complex subunit alpha(NACA) | gi\|209736954 | 4.6e-10 | -1.88 | - | - | - | - | 155 | 4 |
| 33-50 | Aldo-keto reductase family 1 member D19(AKR1D1) | gi\|118082901 | 0.00011 | -1.57 | - | - | - | - | 101 | 6 |
| 34-05 | Heat shock 70 kDa protein 8 (HSPA8) | gi\|190576828 | 0.004 | 2.28 | - | - | - | - | 76 | 5 |
| 34-06 | cleavage stimulation factor, subunit 2 (CSTF2) | gi\|224097289 | 0.0017 | 5.28 | - | - | - | - | 89 | 5 |
| 34-09 | Vimentin(VIM) | gi\|114326309 | 5.8e-32 | 3.41 | - | - | - | - | 374 | 15 |
| 34-10 | actin, cytoplasmic type 5(ACTG1) | gi\|56119084 | 9.1e-18 | 2.49 | 9.1e-18 | 3.63 | - | - | 232 | 4 |
| 34-13 | serine/threonine-protein phosphatase PP1-gamma catalytic subunit(PPP1CC) | gi\|57525187 | 2.9e-19 | 7.21 | 2.9e-19 | 5.86 | - | - | 247 | 12 |
| 34-15 | Vimentin(VIM) | gi\|57240090 | 3.6e-37 | 3.57 | 3.6e-37 | 3.75 | - | - | 426 | 15 |
| 34-16 | vimentin(VIM) | gi\|57240090 | 7.2e-20 | 3.64 | - | - | - | - | 253 | 10 |
| 34-17 | 14-3-3protein beta/alpha (YWHAB) | gi\|57529350 | 9.1e-08 | 2.04 | - | - | - | - | 132 | 7 |
| 34-18 | lamin-B2(LMNB2) | gi\|45384202 | 9.1e-16 | 3.66 | 9.1e-16 | 3.03 | - | - | 212 | 11 |
| 34-19 | calpain small subunit（CAPNS1） | gi\|2506056 | 3.6e-28 | 100.21 | - | - | 3.6e-28 | 100 | 336 | 3 |
| 34-21 | Vimentin (VIM) | gi\|57240089 | 3.6e-19 | 2.64 | 3.6e-19 | 2.50 | - | - | 246 | 7 |
| 34-26 | chloride intracellular channel protein 2（CLIC2） | gi\|71895359 | 7.2e-13 | 25.99 | - | - | 7.2e-13 | -100 | 183 | 4 |
| 34-28 | calpain small subunit  （CAPNS1） | gi\|2506056 | 1.1e-05 | 24.55 | - | - | 1.1e-05 | -100 | 111 | 2 |
| 34-29 | glyceraldehyde-3-phosphate dehydrogenase  (GAPDH) | gi\|63401 | 3.6e-13 | 18.52 | - | - | 3.6e-13 | -1.78 | 186 | 3 |
| 34-33 | Vimentin(VIM) | gi\|114326309 | 5.8e-13 | 2.06 | 5.8e-13 | 1.87 | - | - | 184 | 10 |
| 35-12 | cytoskeleton-associated protein 4(CKAP4) | gi\|118082813 | - | - | 5.8e-15 | -4.69 | 5.8e-15 | 8.63 | 204 | 8 |
| 35-14 | keratin, type I cytoskeletal 19(KRT19) | gi\|45384356 | - | - | 5.8e-06 | -3.19 | 5.8e-06 | 3.49 | 114 | 10 |
| 35-18 | cathepsin D precursor(CTSD) | gi\|45384002 | - | - | 1.8e-05 | -2.45 | - | - | 109 | 2 |
| 35-20 | septin-2(SEPT2) | gi\|57525156 | - | - | 5.8e-30 | -2.11 | - | - | 354 | 8 |
| 35-21 | heterogeneous nuclear ribonucleoprotein C (C1/C2), isoform CRA_c(HNRPC) | gi\|119586801 | - | - | 0.0044 | -1.65 | - | - | 85 | 2 |
| 36-03 | lamin-A(LMNA) | gi\|45384214 | - | - | 2.9e-25 | 1.68 | - | - | 307 | 22 |
| 36-05 | Gallus gallus aldehyde dehydrogenase 7 family, member A1  (ALDH7A1) | gi\|118104602 | - | - | 0.015 | 100 | 1.8e-17 | 2.17 | 80 | 5 |
| 36-11 | F-actin-capping protein subunit alpha-1  (CAPZA1) | gi\|297787503 | - | - | 1.8e-17 | 3.05 | - | - | 229 | 8 |
| 36-13 | Putative methyltransferase 24 (3D495) | gi\|50750103 | - | - | 2.3e-11 | 100 | 2.3e-11 | -3.47 | 168 | 6 |
| 36-14 | pyruvate dehydrogenase E1 component subunit beta(PDHB) | gi\|310750374 | - | - | 1.1e-16 | 1.78 | - | - | 221 | 5 |
| 36-18 | calpain small subunit（CAPNS1） | gi\|2506056 | - | - | 1.1e-07 | 1.73 | 1.1e-07 | -2.17 | 131 | 3 |

a to g refer to the corresponding footnotes in table 1.
